# Osmotic Tension Asymmetry Drives Electrotactic Migration via PDLIM7‐Polarized Microfilament Coordination in Breast Cancer Cells

**DOI:** 10.1002/advs.202515246

**Published:** 2025-12-22

**Authors:** Ling Zhu, Zihui Zheng, Wang Li, Chenyi Shou, Ying Zhao, Lijun Zhang, Xiaoli Shi, Yunfeng Hu, Huanhuan Zhao, Huiwen Wu, Jun Guo

**Affiliations:** ^1^ State Key Laboratory on Technologies for Chinese Medicine Pharmaceutical Process Control and Intelligent Manufacture Nanjing University of Chinese Medicine Nanjing P. R. China; ^2^ Department of Biochemistry and Molecular Biology School of Medicine Nanjing University of Chinese Medicine Nanjing Jiangsu P. R. China; ^3^ Basic Medical Experiment Center, School of Medicine Nanjing University of Chinese Medicine Nanjing Jiangsu P. R. China; ^4^ Laboratory Center for Basic Medical Sciences Nanjing Medical University Nanjing P. R. China

**Keywords:** breast cancer, directional migration, electrotaxis, osmotic pressure asymmetry, PDLIM7

## Abstract

Directional migration of tumor cells depends on mechanical forces generated by intracellular asymmetry. This study establishes an electric field‐induced directional migration model using fluorescence tension probes to visualize microfilament forces and intracellular osmotic pressure dynamics. Electric fields induce protein kinase A (PKA)‐dependent PDLIM7 recruitment, polarizing microfilament tension at cell edges via site‐specific phosphorylation at serine 190. Concurrently, the electric field induces asymmetric changes in membrane potential, driven by protein nanoparticles and calcium ions, regulating osmotic tension. Leading‐edge depolarization activates TMEM16A channel, while trailing‐edge hyperpolarization activates the small conductance calcium‐activated potassium (SK) channel. Chloride influx and potassium efflux create differential ion diffusion, resulting in leading‐edge hypertonic expansion and trailing‐edge contraction, thereby dictating the directionality of electrotactic migration. Osmotic pressure asymmetry further modulates PKA polarity, amplifying directional migration cues. This study elucidates the coordinated interplay between osmotic tension and membrane potential in cellular electromechanics, revealing a mechanistic framework where osmotic tension asymmetry orchestrates tumor cell migration through polarized PDLIM7‐microfilament tension regulation.

## Graphical abstract

This study establishes an electric field‐induced directional migration model using fluorescence tension probes to visualize microfilament forces and intracellular osmotic pressure dynamics in the electrotactic migration of breast cancer cells. This model delineates how electromechanical interactions among membrane potential, ion channels, OP, traction proteins, and cytoskeletal tension coordinate tumor cell migration within the electric field microenvironment.

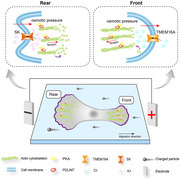

## 1 Introduction

Cell electrotaxis, characterized by directional migration under an external electric field, serves as a pivotal driving force in tumor metastasis and other pathological processes [1, 2]. Electrotaxis regulates cellular positioning within diverse physiopathological environments, such as with electric field stimuli inducing reorientation of cell surface structures and signaling molecules [3]. External electric fields modulate cell membrane properties, initiating polarized cell dynamics that culminate in cytoskeletal reorganization and cellular polarity [4, 5]. The polarization and imbalance of intracellular tension are closely linked to the directional migration of tumor cells [6, 7].

PDZ (postsynaptic density 95‐discs large‐zonula occludens 1) and LIM (Lin‐11, Isl‐1, and Mec‐3) domain protein 7 (PDLIM7) is a critical microfilament‐binding protein involved in cytoskeletal dynamics [8, 9], The N‐terminal PDZ domain of PDLIM7 mediates interactions with transmembrane proteins, while its intrinsically disordered region (IDR) facilitates phase separation [10]. The C‐terminal mechanosensitive LIM domain binds directly to F‐actin in a force‐dependent manner. PDLIM7 localizes to focal adhesions and plays a significant role in tumor cell migration and invasion [10]. Previous studies have demonstrated that the LIM domain undergoes conformational changes under mechanical stress, functioning as a mechanical response module [11, 12, 13]. PDLIM7 is highly expressed in various cancers, such as prostate cancer, thyroid cancer, and clear cell renal cell carcinoma. Regarding the potential of therapeutic targets, knocking down PDLIM7 can significantly inhibit the proliferation, migration and invasion of tumor cells, and enhance the sensitivity of tumor cells to chemotherapeutic drugs (such as docetaxel), suggesting the potential of direct therapeutic target of PDLIM7 [14]. While it is hypothesized that microfilament tension polarization is achieved through PDLIM7‐mediated force transduction, whether PDLIM7 dictates the directionality of cell migration remains a subject of debate.

Osmosis, driven by water permeation, also represents a fundamental force in cell migration, governed by the regulation of cell volume and ion‐water flux across the membrane [15, 16, 17]. The polarized distribution of Na^+^/H^+^ exchangers and aquaporins facilitates net inflow of water and ions at the leading edge and net outflow at the trailing edge, promoting cellular displacement [18]. However, the movement of charged ions necessitates consideration of membrane potential effects [19]. Notably, under isotonic extracellular conditions, it remains unclear whether intracellular osmotic pressure (OP) imbalances exist. Assessing osmotic tension differences requires precise mechanical traction measurements.

Osmotic tension is intricately associated with ion channel activation, permeability, and diffusion [19]. Multiple sodium, chloride, and Gardos channels contribute to cell swelling and shrinkage responses [21]. Interestingly, many of these channels are both voltage‐dependent and calcium‐activated. In electrostimulated environments, alterations in membrane potential can disrupt the distribution of ion channels, leading to ion concentration gradients [20]. Changes in OP can dynamically remodel the cytoskeletal network, regulate transmembrane water flux, and influence [21] cell morphology [22]. Intracellular hyperosmotic conditions are often accompanied by depolymerization of microfilaments and microtubules. OP gradients across the cell alter cytoplasmic flow direction, driving polarized organization of the microfilament network [23]. Our previous studies have found microfilament depolymerization induced production of massive intracellular protein nanoparticles (PNs) and Ca^2+^ increase, both involved in the osmotic pressure regulation. Ca^2+^ content is involved in the membrane potential transition between depolarization and hyperpolarization in a PNs‐dependent manner [21, 24]. In addition, localized activation of osmotic‐sensitive signaling molecules, such as protein kinases and calmodulin, modulates actin‐binding protein activity, thereby influencing pseudopodia formation at the leading edge and contraction at the trailing edge [25, 26]. Therefore, it is essential to determine if an imbalanced distribution of osmotic tension, characterized by leading‐edge expansion and trailing‐edge contraction, is responsible for directional cell migration.

In this study, an electrotaxis model of tumor cells is established, integrating visual tension detection through a fluorescence resonance energy transfer (FRET)‐based PDLIM7 tension probe. Directionally migrating cells exhibit asymmetric osmotic tension, with hyperosmotic and hypoosmotic regions driving polarized recruitment of PKA and PDLIM7, as well as microfilament tension polarization at the leading and trailing edges. This osmotic tension asymmetry is induced by an unbalanced distribution of membrane potential, regulated by protein nanoparticles and calcium ions. Activation of distinct ion channels and ion diffusion pathways synergistically orchestrates differential distributions of osmotic tension and membrane potential. These findings suggest that electromechanical coupling‐mediated osmotic tension imbalance governs tumor cell migration and dictates migration directionality.

## 2 Results

### 2.1 Electric Fields Drive PDLIM7 Recruitment at the Cell Leading and Trailing Edges and Enhance Cell Migration

**FIGURE 1:**
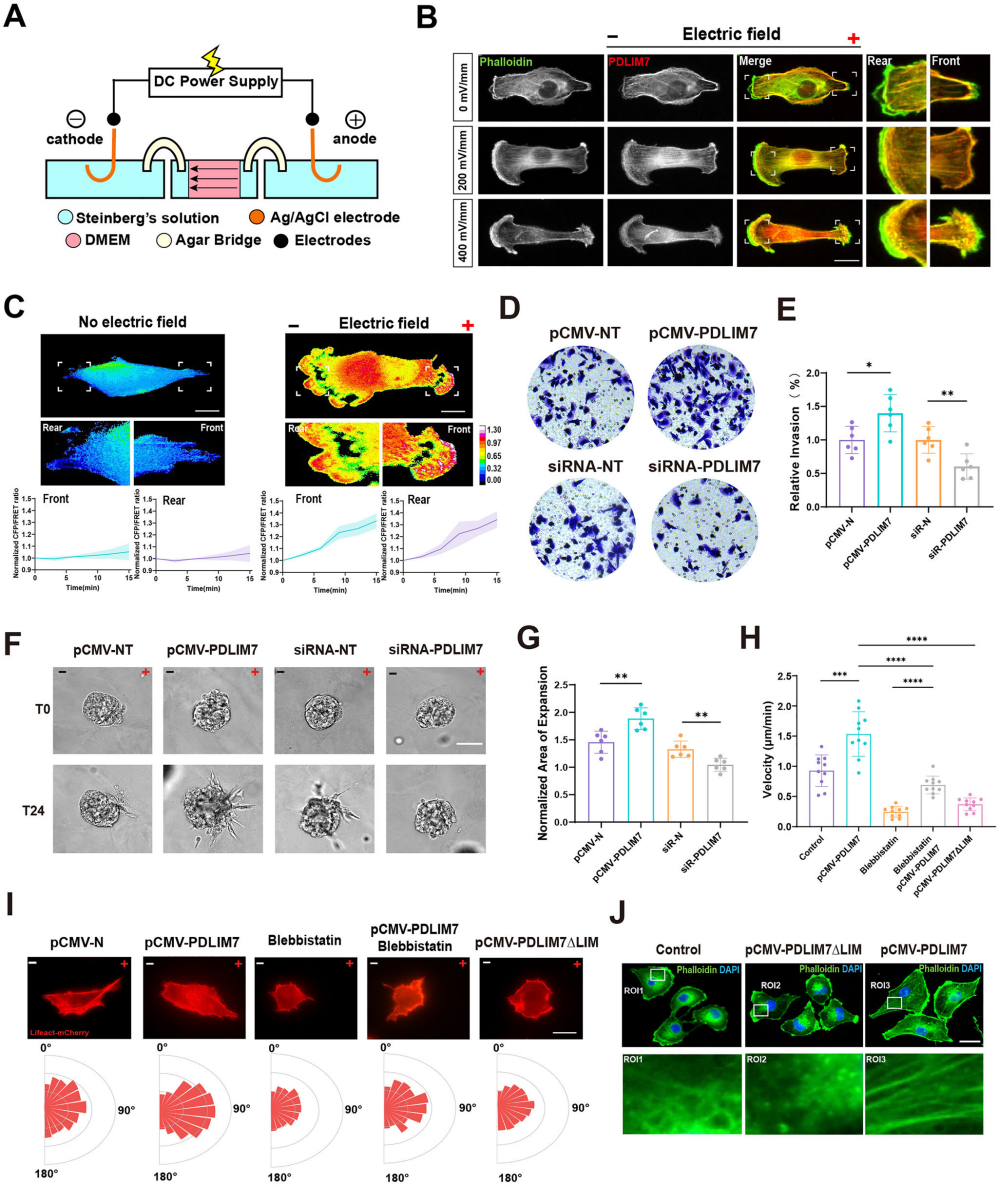
Direct current electric fields regulate PDLIM7 polarization and promote breast cancer cell migration. (A) Schematic cross‐section of the electro‐bioreactor. (B) Representative fluorescence images showing the distribution of F‐actin and PDLIM7 in cells subjected to no current, low current (200 mV/mm), and high current (400 mV/mm) stimulation for 1 hour. Green: FITC‐labeled F‐actin; Red: TRITC‐labeled PDLIM7. Scale bar: 10 µm. (C) Top: Time‐lapse FRET images of MDA‐MB‐231 cells expressing the PDLIM7‐M‐cpstFRET probe under a 400 mV/mm electric field for 15 min. Bottom: Normalized CFP/FRET ratios at the cell front and rear (mean ± SD, *n* ≥ 6 cells). Scale bar: 10 µm. Calibration bar: 0.00–1.30. (D,E) Transwell invasion assay of MDA‐MB‐231 cells transfected with pCMV‐PDLIM7 plasmid or PDLIM7‐siRNA. Quantification of invaded cells (mean ± SD, *n* = 6). Statistical analysis: unpaired Student's *t*‐test (^*^ *P* < 0.05, ^**^ *P* < 0.01). (F, G) Dissemination of cells from spheroids embedded in 3D collagen gels, transfected as indicated, at 0 and 24 h. Quantification of cell expansion (mean ± SD, *n* = 6). Unpaired Student's *t*‐test (^**^ *P* < 0.01). (H) Forward migration velocity (µm/min) during electrotaxis over 1 h (mean ± SEM, *n* ≥ 15 cells). One‐way ANOVA was used for comparisons (^***^ *P* < 0.001, ^****^ *P* < 0.0001). (I) Top: Representative images of Lifeact‐transfected cells during electrotaxis (400 mV/mm). Bottom: Rose plots showing orientation angles; bar magnitudes indicate the fraction of cells in each trajectory angle bin (n ≥ 10 cells). Scale bar: 10 µm. (J) Fluorescence images of F‐actin (green, Phalloidin) and nuclei (blue, DAPI) in MDA‐MB‐231 cells. Scale bar: 10 µm.

To investigate whether endogenous electric fields influence breast cancer cell migration, MDA‐MB‐231 cells were stimulated with a direct current (DC) electric field (200 and 400 mV/mm) using a modified bioreactor system (Figure 1A). Small physiological DC electric fields‌ exist in living organisms (e.g., at tumors, wounds, and regeneration sites), providing guidance cues for cell electrotaxis migration [27, 28]. According to previous research, 200–400 mV/mm‌ was applied to screen electrotaxis phenotypes, demonstrating that this strength was sufficient to elicit robust responses and directional migration [29, 30, 31]. Time‐lapse imaging and cell tracking were performed to monitor individual cell migration dynamics, visualized through hairline plots comparing non‐stimulated cells and those exposed to electric field stimulation. In the absence of an electric field, MDA‐MB‐231 cells exhibited random migration, dispersing radially. However, upon electric field application, these cells displayed persistent anodal migration (Figure S1A,B). Furthermore, migration velocity increased in a field strength‐dependent manner (Figure S1C). Moreover, electric field exposure induced a more polarized phenotype in MDA‐MB‐231 cells, as evidenced by a significant decrease in cell circularity compared to control cells (Figure S1D).

Analysis of The Cancer Genome Atlas (TCGA) datasets revealed that elevated PDLIM7 expression correlates with high‐grade, aggressive tumors (Figure S2A). Kaplan‐Meier survival analysis demonstrated that patients with high PDLIM7 expression had poorer overall survival (Figure S2B). We next explored whether PDLIM7 directly modulates the invasive capacity of triple‐negative breast cancer (TNBC) cells in vitro. Immunofluorescence imaging showed that PDLIM7 and F‐actin were recruited to cellular protrusions at the leading edge and lamellar pseudopodia at the trailing edge, with recruitment intensity increasing in response to electric fields ranging from 0 to 400 mV mm^−1^ (Figure 1B).

To visualize real‐time changes in PDLIM7 tension, a fluorescence resonance energy transfer (FRET)‐based tension probe was constructed (Figure S3A). Upon photobleaching, donor fluorescence (eCFP) increased due to reduced energy transfer efficiency, yielding a FRET after‐bleaching (FRET AB) of 3.37% (Figure S3B). Time‐lapse imaging over 200 s was conducted, and fluorescence recovery within regions of interest (ROI) reached 85.35% at 500 s post‐bleaching (Figure S3C). To validate the probe's sensitivity to cytoskeletal tension, hypotonic buffer treatment was applied. Cells with PDLIM7 knockdown and a re‐expressed tension probe were subjected to 15 min of time‐lapse imaging, which revealed a progressive increase in PDLIM7‐M‐cpstFRET tension loading. Importantly, no FRET ratio changes were observed in PDLIM7 force‐insensitive control probes (Figure S3D–F). These findings confirmed the probe's capability to report real‐time PDLIM7 tension during cell migration. Interestingly, applying a 400 mV mm^−1^ electric field for 15 min significantly increased PDLIM7 tension, exhibiting polarization at both the leading and trailing edges of MDA‐MB‐231 and MDA‐MB‐468 cells (Figure 1C; Figure S4A). However, in low‐invasive MCF‐7 cells, PDLIM7 polarization was not observed under electric field stimulation (Figure S4B).

To further assess PDLIM7's role in TNBC cell invasion, MDA‐MB‐231 cells were transfected with either a PDLIM7 overexpression plasmid or PDLIM7‐targeting small interfering RNA (siRNA) (Figure S1E). Transwell migration assays demonstrated a positive correlation between PDLIM7 expression levels and cell migratory capacity (Figure 1D,E). To extend these findings in a physiologically relevant context, the invasive behavior of breast cancer spheroids embedded in 3D collagen gels was evaluated. Spheroids composed of PDLIM7‐knockdown cells exhibited a significantly reduced area of dissemination upon electric field stimulation, indicative of a less invasive phenotype (Figure 1F,G). Further experiments revealed that pharmacological inhibition of myosin II with blebbistatin, as well as deletion of the PDLIM7 LIM domains, effectively abolished microfilament tension and impaired directed migration, fully antagonizing PDLIM7‐induced anodal migration (Figure 1H,I). Given that the LIM domain is essential for F‐actin binding, truncation of this domain markedly reduced the number of force‐activated F‐actin filaments (Figure 1J). These results support a model in which electric field‐induced recruitment of PDLIM7 at the leading and trailing edges establishes cell polarity and promotes migration toward the anode.

### 2.2 PKA‐Mediated Ser190 Phosphorylation of PDLIM7 IDR Domain Regulates Tension Polarization during Electrotactic Migration

The IDR of LIM domain scaffolding proteins has been reported to drive phase separation through multivalent interactions, contributing to focal adhesion (FA) maturation, with phosphorylation modulating phase separation via electrostatic effects [32]. This prompted us to investigate whether the force transmission efficiency of PDLIM7 is regulated by phosphorylation of its IDR domain.

**FIGURE 2:**
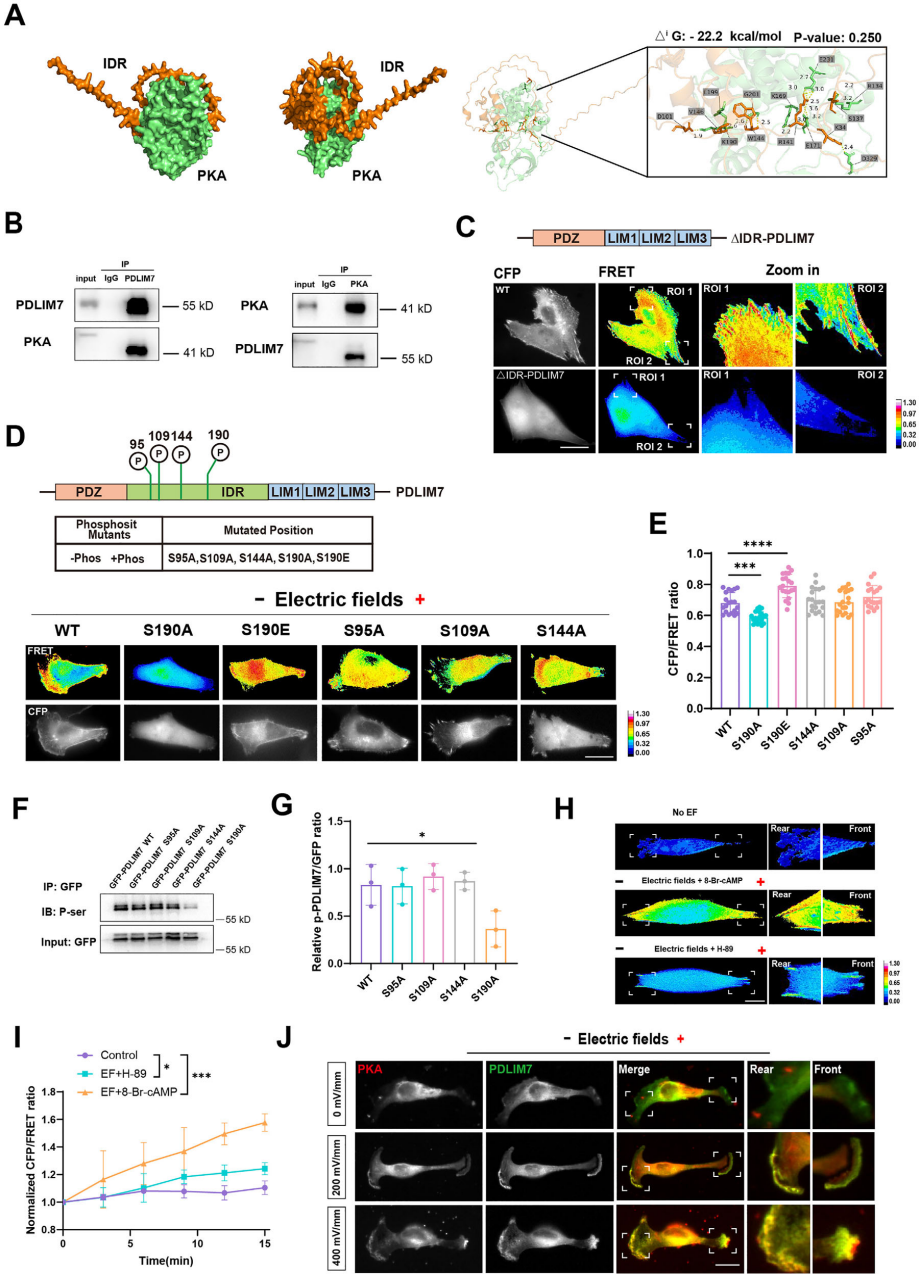
Ser190 phosphorylation in the IDR domain regulates PDLIM7 tension. (A) Left: AlphaFold2‐predicted structure of PDLIM7 binding to PKA. Orange: PDLIM7 IDR domain; Green: PKA. Right: Enlarged view of the binding interface with affinity values. (B) Immunoprecipitation showing endogenous PKA interaction with PDLIM7 in fresh human TNBC tissues. (C) Representative FRET images of MDA‐MB‐231 cells expressing PDLIM7‐M‐cpstFRET or PDLIM7△IDR‐M‐cpstFRET probes. Scale bar: 10 µm. (D) FRET images of cells expressing PDLIM7 variants with specific Ser/Ala or Ser/Glu mutations (S95A, S109A, S144A, S190A, S190E) under 400 mV/mm stimulation. Scale bar: 10 µm. Calibration bar: 0.00–1.30. (E) Quantification of normalized CFP/FRET ratios (mean ± SEM, *n* ≥ 20 cells). One‐way ANOVA (^***^ *P* < 0.001, ^****^ *P* < 0.0001). (F) Western blot analysis of phosphorylated PDLIM7 in cells expressing WT or mutant GFP‐PDLIM7 constructs. (G) Quantification of phosphorylated PDLIM7/input ratio (*n* ≥ 3). One‐way ANOVA (^*^ *P* < 0.05). (H) Representative FRET images of cells treated with PKA inhibitors/activators under 400 mV/mm stimulation. Scale bar: 10 µm. Calibration bar: 0.00–1.30. (I) Quantification of normalized CFP/FRET ratios (mean ± SEM, *n* ≥ 6 cells). One‐way ANOVA (^*^ *P* < 0.05, ^***^ *P* < 0.001). (J) Immunofluorescence of endogenous PKA and PDLIM7 in cells exposed to 0, 200, or 400 mV/mm fields. Green: FITC‐labeled PDLIM7; Red: TRITC‐labeled PKA. Scale bar: 10 µm.

Bioinformatic analysis predicted that serine 190 (Ser190) of PDLIM7 is a potential phosphorylation site for PKA [33]. Molecular docking simulations further revealed a strong binding affinity between the PDLIM7 IDR domain and PKA, with a binding energy of −22.2 kcal/mol, significantly higher than that of the PDZ or LIM domains (Figure 2A; Figure S5A). Co‐immunoprecipitation (co‐IP) assays confirmed a robust interaction between PDLIM7 and PKA in MDA‐MB‐231 cells (Figure 2B). Additionally, deletion of the IDR domain (ΔIDR) markedly reduced PDLIM7 tension and impaired the formation of cellular pseudopodia (Figure 2C), underscoring the critical role of the IDR in force transmission.

To further elucidate the role of IDR phosphorylation, we generated a phosphorylation‐deficient mutant (–Phos) by mutating four phosphorylation sites within the IDR to alanine, and a phosphorylation‐mimetic mutant (+Phos) by substituting Ser190 with glutamic acid (Figure 2D). A series of FRET‐based PDLIM7 tension probes, including four phosphorylation‐deficient mutants (PDLIM7‐S95A, PDLIM7‐S109A, PDLIM7‐S144A, and PDLIM7‐S190A) and a phosphorylation‐mimetic mutant (PDLIM7‐S190E), were constructed to investigate site‐specific phosphorylation effects on tension transmission. FRET analysis demonstrated that the PDLIM7‐S109A mutant exhibited significantly reduced tension, which was essential for establishing tension polarization at the cell front and rear (Figure 2D,E). Furthermore, immunoprecipitation assays revealed that cells expressing PDLIM7‐S190A displayed a substantial decrease in phosphorylated PDLIM7 (p‐PDLIM7) levels, whereas the other three mutants maintained phosphorylation levels comparable to the wild‐type protein (Figure 2F,G). To ensure the experimental conclusions are more reliable, we performed further assays of the migration behavior of PDLIM7‐S109A, PDLIM7‐S95A, PDLIM7‐S190A and PDLIM7‐S144A mutants, including cell migration trajectories and quantification of migration velocity. Time‐lapse imaging and cell tracking results showed that only PDLIM7‐S190A shortened migration trajectories and decreased migration velocity (Figure S7A,B), thereby excluding other PKA phosphorylation sites.

These findings collectively indicate that Ser190 phosphorylation within the PDLIM7 IDR domain is crucial for regulating its tension polarization during electrotactic migration. To further delineate the relationship between PDLIM7 tension and PKA activity under electric field stimulation, we treated MDA‐MB‐231 cells with 8‐Br‐cAMP, a PKA agonist. This treatment significantly increased cellular tension, particularly at the leading and trailing edges. Conversely, inhibition of PKA activity using H‐89 led to a pronounced reduction in cellular tension (Figure 2H,I). We further examined PDLIM7 tension after treatment with inhibitors of other kinases (e.g., PKC, CaMKII), but the results showed that the PDLIM7 tension did not decrease, indicating the poor possibility of binding between PDLIM7 and PKC/CaMKII (Figure S8A–C). Immunofluorescence assays further demonstrated that PKA and PDLIM7 colocalized at the leading and trailing edges, with cell polarity and fluorescence intensity progressively increasing in response to stronger electric fields (Figure 2J). These results collectively suggest that PKA activity is essential for PDLIM7 polarization at the cell periphery, facilitating tension polarization during electrotactic migration.

### 2.3 Ser190 Phosphorylation of PDLIM7 Suppresses TNBC Cell Invasion and Metastasis In Vitro and In Vivo

**FIGURE 3:**
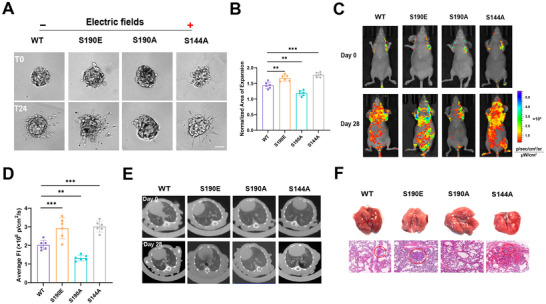
PDLIM7 Ser190 phosphorylation promotes TNBC invasion in vitro and in vivo. (A) Dissemination of MDA‐MB‐231 spheroids transfected with PDLIM7‐WT, S190A, S190E, or S144A constructs embedded in 3D collagen gels at 0 and 24 h under 400 mV/mm stimulation. Scale bar: 100 µm. (B) Quantification of cell expansion (mean ± SD, *n* = 6). One‐way ANOVA (^**^ *P* < 0.01, ^***^ *P* < 0.001). (C) Representative bioluminescence images of systemic metastases in nude mice at 0 and 28 days post‐injection. (D) Quantification of fluorescence intensity in tumor regions (mean ± SD, *n* = 6). One‐way ANOVA (^**^ *P* < 0.01, ^***^ *P* < 0.001). (E) Orthotopic implantation assay showing CT images. (F) Representative lung tissues, and H&E‐stained lung sections. Red circles indicate metastatic nodules on the 28th day.

To further validate the physiological relevance of Ser190‐dependent PDLIM7 tension in tumor cell invasion, we assessed cell dissemination from 3D breast cancer spheroids embedded in collagen matrices. PDLIM7 knockdown MDA‐MB‐231 cells were transfected with plasmids encoding wild‐type PDLIM7 (PDLIM7‐WT), a phosphorylation‐deficient mutant (PDLIM7‐S190A), a phosphorylation‐mimetic mutant (PDLIM7‐S190E), and a control mutant (PDLIM7‐S144A). Consistent with single‐cell observations, the PDLIM7‐S190A group significantly impaired MDA‐MB‐231 cell dissemination from spheroids compared to the PDLIM7‐S190E and PDLIM7‐S144A groups (Figure 3A). To quantify local invasiveness, the expansion area of spheroids was measured after 24 h of embedding in 3D collagen gels. Spheroids composed of PDLIM7‐S190A cells exhibited a markedly reduced expansion area, indicative of a less invasive phenotype (Figure 3B).

Given the critical role of PDLIM7 phosphorylation in spheroid dissemination in vitro, we next evaluated its functional contribution to breast cancer metastasis in vivo. MDA‐MB‐231 cells expressing PDLIM7‐WT, PDLIM7‐S190A, PDLIM7‐S190E, or PDLIM7‐S144A were orthotopically injected into the fourth mammary fat pad of BALB/c mice. Fluorescence imaging revealed significantly reduced spontaneous metastatic signals in the PDLIM7‐S190A group compared to the other groups (Figure 3C,D). Micro‐computed tomography (micro‐CT) analysis indicated a higher incidence of lung metastases in the PDLIM7‐S190E group, as evidenced by pronounced pulmonary lesions and increased intrapulmonary nodules, corroborated by lung gross anatomy and hematoxylin‐eosin (H&E) staining (Figure 3E,F). No detectable metastases were observed in other organs, including the heart, liver, spleen, kidneys, and brain (Figure S6A). Furthermore, survival analysis demonstrated that mice in the PDLIM7‐S190A group exhibited significantly prolonged survival compared to the other groups (Figure 3F). Collectively, these in vivo findings align with the in vitro data, highlighting the pivotal role of PDLIM7 Ser190 phosphorylation in promoting the invasion and metastasis of triple‐negative breast cancer (TNBC) cells.

### 2.4 Electric Fields Depolarize the Cell Leading Edge and Hyperpolarize the Trailing Edge, Mediating Front and Rear Osmotic Pressure Regulation during Electrotactic Migration

PKA functions as a stress‐responsive kinase regulated by osmotic stimuli [34]. Direct current (DC) electric fields naturally influence ion influx and distribution, resulting in alterations in cell membrane potential and osmotic pressure [35]. To determine whether osmotic pressure modulates PKA distribution, cells were exposed to varying osmotic conditions (hypotonic: 180 mOsm/L, isotonic: 300 mOsm/L, hypertonic: 600 mOsm/L). Immunofluorescence assays revealed marked colocalization of PKA and PDLIM7 at the cell periphery following both hypotonic and hypertonic shocks (Figure S9A). Co‐immunoprecipitation (co‐IP) further confirmed a significant increase in phosphorylated PDLIM7 (p‐PDLIM7) under hypotonic and hypertonic conditions, while isotonic treatment‐maintained phosphorylation levels comparable to the wild‐type group (Figure S9B,C). Additionally, PKA activity and intracellular cyclic AMP (cAMP) content were elevated under hypo‐ and hypertonic stress compared to isotonic controls (Figure S9D,E). FRET‐based tension analysis indicated that PDLIM7 tension was enhanced and localized at the cell edges following osmotic shock, accompanied by cell swelling under hypotonic and shrinkage under hypertonic conditions (Figure S9F,G).

**FIGURE 4:**
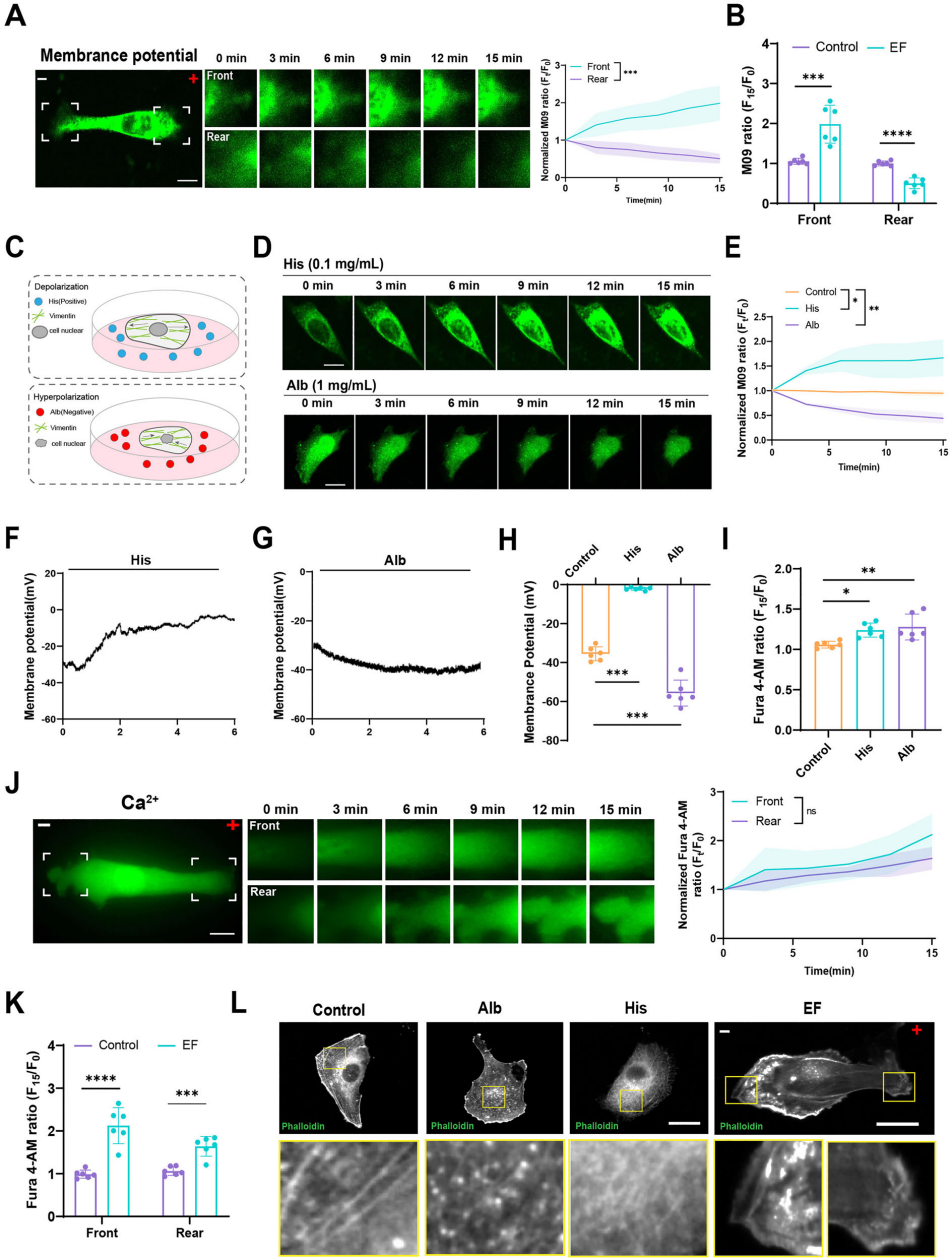
Electric fields induce depolarization at the leading edge and hyperpolarization at the trailing edge of MDA‐MB‐231 cells. (A) Left: Time‐lapse images of membrane potential‐sensitive dye after 15 min of electric field exposure (400 mV/mm). Right: Quantification of M09 fluorescence intensity at the leading and trailing edge of MDA‐MB‐231 cells. (mean ± SEM, *n* ≥ 6 cells). Unpaired Student's *t*‐test (^***^ *P* < 0.001). Increased fluorescence indicates depolarization; decreased fluorescence indicates hyperpolarization. Scale bar: 10 µm. (B) Quantification of M09 fluorescence intensity at the leading and trailing edge of MDA‐MB‐231 cells after treatment with control and electric field. (mean ± SEM, *n* ≥ 6 cells). Unpaired Student's *t*‐test (^***^ *P* < 0.001, ^****^ *P* < 0.0001). (C) Schematic diagram of treatment with histone (0.1 mg/mL) and albumin (1 mg/mL). (D) Membrane potential changes after treatment with histone (0.1 mg/mL) and albumin (1 mg/mL) for 15 min. Scale bar: 10 µm. (E) Quantification of fluorescence intensity (mean ± SEM, *n* ≥ 6 cells). One‐way ANOVA (^*^ *P* < 0.05, ^**^ *P* < 0.01). (F,G) Whole‐cell patch‐clamp trace maps of membrane potential. (H) Membrane potential changes post‐treatment. (mean ± SEM, *n* ≥ 6). One‐way ANOVA (^***^ *P* < 0.001). (I) Quantification of Ca^2^⁺ signals post‐treatment (mean ± SEM, *n* ≥ 6). One‐way ANOVA (^*^ *P* < 0.05, ^**^ *P* < 0.01). (J) Left: Time‐lapse Ca^2^⁺ imaging under 400 mV/mm electric field stimulation (mean ± SEM, *n* ≥ 6). Right: Quantification of Ca^2^⁺ fluorescence intensity at the leading and trailing edge of MDA‐MB‐231 cells. (mean ± SEM, *n* ≥ 6 cells). Unpaired Student's t‐test (ns: not significant). Scale bar: 10 µm. (K) Quantification of Ca^2^⁺ fluorescence intensity at the leading and trailing edge of MDA‐MB‐231 cells after treatment with control and electric field (mean ± SEM, *n* ≥ 6 cells). Unpaired Student's *t*‐test (^***^ *P* < 0.001, ^****^ *P* < 0.0001). (L) Representative images of F‐actin in MDA‐MB‐231 cells post‐treatment. Phalloidin‐stained microfilament images. Scale bar: 10 µm.

Previous studies have demonstrated that electric fields modulate membrane potential at the cell poles, influencing ion flux across the plasma membrane [36]. To investigate this further, we assessed membrane potential dynamics in MDA‐MB‐231 and MDA‐MB‐468 cells using a voltage‐sensitive fluorescent dye. The leading edge (facing the anode) displayed increased fluorescence, indicating depolarization, while the trailing edge (facing the cathode) exhibited decreased fluorescence, indicating hyperpolarization (Figure 4A,B; Figure S11D,E).

To mimic anode and cathode effects independently, albumin (negatively charged) and histone (positively charged) were applied to cells, simulating cathodal and anodal environments, respectively (Figure 4C; Figure S10A). In line with electric field observations, histone induced depolarization and albumin induced hyperpolarization in MDA‐MB‐231 cells (Figure 4D,E). These findings were corroborated by patch‐clamp recordings, confirming membrane potential changes consistent with dye‐based imaging (Figure 4F–H).

Our previous studies suggested that calcium ions (Ca^2^⁺) are integral to membrane potential transitions between depolarization and hyperpolarization in a protein nanoparticle (PN)‐dependent manner. In this study, calcium imaging revealed increased Ca^2^⁺ concentrations at both the leading and trailing edges of MDA‐MB‐231 cells under electric field stimulation, which was similarly observed in MDA‐MB‐468 cells (Figure 4I–K; Figure S11F–G).

In addition, we observed significant microfilament depolymerization at the trailing edge following electric field exposure, whereas depolymerization at the leading edge was comparatively minimal in both MDA‐MB‐231 and MDA‐MB‐468 cells (Figure 4L; Figure S11A). Quantification of intracellular protein nanoparticles under albumin and histone treatments showed that negatively charged albumin environments promoted greater nanoparticle formation (Figure S11B,C).

**FIGURE 5:**
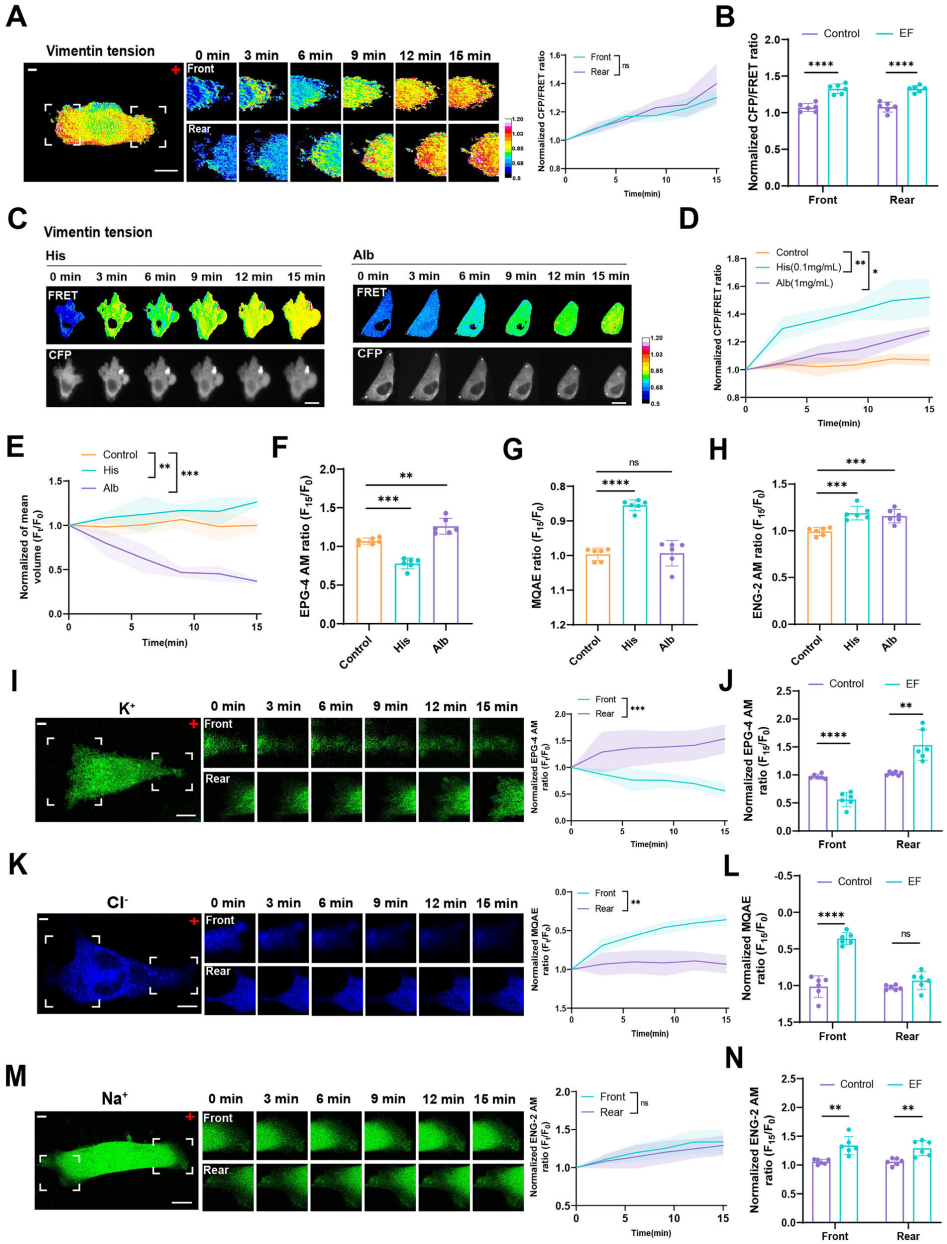
Electric fields regulate osmotic pressure dynamics at the front and rear of MDA‐MB‐231 cells. (A) Left: FRET time‐lapse images of Vimentin‐M‐cpstFRET expressing cells under 400 mV/mm stimulation. Scale bar: 10 µm. Right: Normalized CFP/FRET ratios at the leading and trailing edge of MDA‐MB‐231 cells. (mean ± SEM, *n* ≥ 6 cells). Unpaired Student's *t*‐test (ns: not significant). (B) Normalized CFP/FRET ratios at the leading and trailing edge of MDA‐MB‐231 cells after treatment with control and electric field. (mean ± SEM, *n* ≥ 6 cells). Unpaired Student's *t*‐test (^****^ *P* < 0.0001). (C) FRET images after histone and albumin treatment. Scale bar: 10 µm. (D) Normalized CFP/FRET ratios post‐treatment. (mean ± SD, *n* ≥ 6). One‐way ANOVA (^*^ *P* < 0.05, ^**^ *P* < 0.01). (E) Mean cell volume changes post‐treatment (mean ± SD, *n* ≥ 6). One‐way ANOVA (^**^ *P* < 0.01, ^***^ *P* < 0.001). (F–H) Quantification of intracellular K⁺, Cl^−^, and Na⁺ concentrations post‐treatment (mean ± SD, *n* ≥ 6). One‐way ANOVA (^**^ *P* < 0.01, ^***^ *P* < 0.001, ^****^ *P* < 0.0001, ns: not significant). (I,K,M) Left: Time‐lapse images of K⁺, Cl^−^, and Na⁺ distribution under 400 mV/mm stimulation. Right: Quantification of K⁺, Cl^−^, and Na⁺ fluorescence intensity at the leading and trailing edge of MDA‐MB‐231 cells. (mean ± SEM, *n* ≥ 6 cells). Unpaired Student's *t*‐test (ns: not significant, ^**^ *P* < 0.01, ^***^ *P* < 0.001). Scale bar: 10 µm. (J, L, N) Quantification of K⁺, Cl^−^, and Na⁺ fluorescence intensity at the leading and trailing edge of MDA‐MB‐231 cells after treatment with control and electric field. (mean ± SEM, *n* ≥ 6 cells). Unpaired Student's t‐test (ns: not significant, ^**^ *P* < 0.01, ^****^ *P* < 0.0001).

According to previous reports, the osmotic tension effect relies on electromechanical coupling mediated by intracellular PN and Ca^2^⁺ levels [21]. To visualize osmotic pressure dynamics under electric fields, a Vimentin‐M‐cpstFRET probe was employed. FRET analysis revealed significantly increased tension loads at both the leading and trailing edges in MDA‐MB‐231 and MDA‐MB‐468 cells upon electric field stimulation and histone/albumin treatments (Figure 5A–D; Figure S11H,I). However, osmotic tension could not change in MCF‐7 cells under the electric fields (Figure S11J,K). Osmotic pressure‐induced changes in cell volume were assessed using 3D live‐cell imaging. Histone treatment induced cellular swelling, while albumin treatment led to cell shrinkage, mimicking electric field‐induced volume alterations (Figure 5E).

Our previous work has reported that the expansive force at the leading edge originates from the inward pulling force of actin filaments [37]. However, the source of the enhanced contractile force at the trailing edge remains unclear. We found that under albumin stimulation, the cell nucleus contracted significantly through laser confocal 3D layer scanning (Figure S12A,B). Therefore, we speculate that the contractile force at the trailing edge may be associated with nuclear deformation. To further verify the relationship between nuclear mechanics and electrotaxis‐induced directional migration, we treated cells with tipifarnib, which disrupts the cell nucleus. After 48 hours of stimuli, we observed nuclear morphological changes, including bubbling or atrophy of the nuclear membrane (Figure S12C). To assess cellular contractility, a gel deformation assay was conducted by quantifying volume changes in collagen gels embedded with cells. Tipifarnib‐treated cells exhibited markedly reduced gel deformation compared to control cells, indicating a marked decline in contractile force (Figure S12D,E). In addition, FRET analysis showed significantly reduced tension of vimentin at the leading edge and trailing edge (Figure S12F,G), indicating that intermediate filament tension changes require the maintenance of dynamic nuclear deformation. These data suggest that the structural and mechanical stability of the nucleus is crucial for maintaining normal cellular contractility and, ultimately, for effective electrotactic migration.

Since cellular volume regulation is largely mediated by Na⁺, Cl^−^, and K⁺ fluxes across membrane channels and transporters, we next analyzed ion flux under electric fields using ion‐specific fluorescent indicators. Notably, Cl^−^ influx was predominantly observed at the leading edge, while K⁺ efflux was enriched at the trailing edge (Figure 5I–L). Additionally, Na⁺ accumulation was detected at both the leading and trailing edges (Figure 5M,N). These findings demonstrate that electric field‐induced Cl^−^‐mediated depolarization at the leading edge and K⁺‐mediated hyperpolarization at the trailing edge coordinate differential osmotic pressures. This asymmetry results in leading‐edge swelling and trailing‐edge contraction, driving directed cell migration under electric fields.

### 2.5 TMEM16A and SK Channels Regulate Osmotic Pressure at the Cell Leading and Trailing Edges during Electrotactic Migration

Ion channels, primarily recognized for their roles in cell polarity and motility programs, have been implicated in mediating electrotaxis in vitro [20]. To determine which ion channels, contribute to osmotic pressure regulation at the leading and trailing edges, we transfected MDA‐MB‐231 cells with a Vimentin‐M‐cpstFRET tension probe and applied chloride (Cl^−^) and potassium (K⁺) channel inhibitors following histone (0.1 mg/mL) and albumin (1 mg/mL) stimulation. Inhibition of Max‐Cl, TMEM16A, and SK channels significantly reduced Vimentin tension at the cell front and rear, respectively (Figure S13A–F). To further exclude other relevant channels, we have performed additional experiments treating electrotaxis‐induced MDA‐MB‐231 cells with other channel inhibitors. FRET results showed inhibition of Piezo1 (Dooku1), aquaporins (TGN‐20) or Na^+^/H^+^ exchangers (EIPA) failed to show the polarized PDLIM7 tension across the cell (Figure S14A–D), suggesting the above channels not involve in the electrotaxis‐induced directional migration. Subsequently, gene knockdown of Max‐Cl, TMEM16A, and SK channels was performed (Figure S15E,F). FRET analysis revealed that PDLIM7 tension decreased markedly in TMEM16A‐knockdown (histone‐stimulated) and SK‐knockdown (albumin‐stimulated) cells, while Max‐Cl knockdown unexpectedly increased PDLIM7 tension (Figure S15A–D), suggesting that TMEM16A and SK channels specifically regulate PDLIM7 tension at the leading and trailing edges, respectively.

**FIGURE 6:**
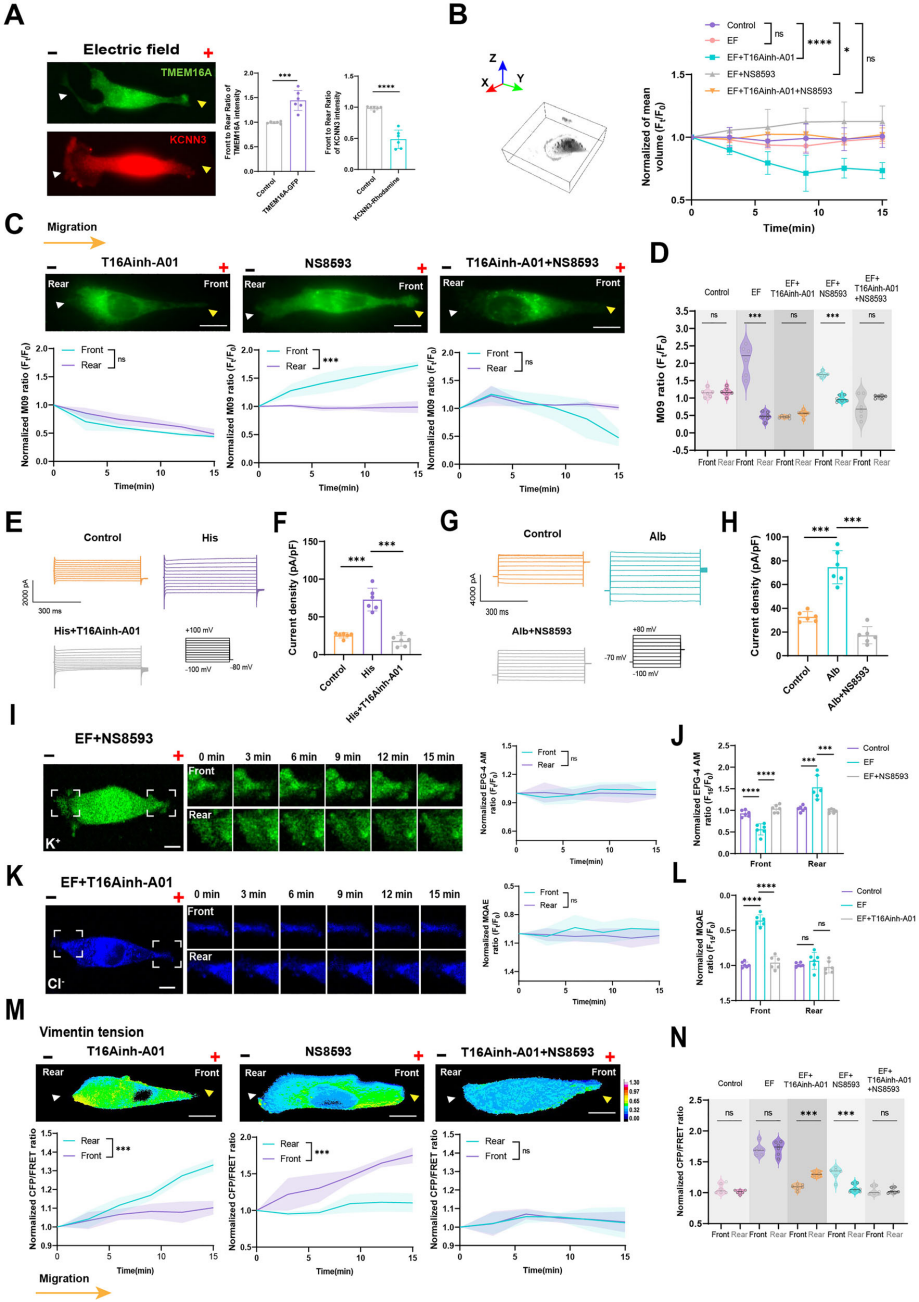
TMEM16A and SK regulate osmotic pressure asymmetry during electrotactic migration. (A) Left: Immunofluorescence images of TMEM16A and SK (KCNN3) under 400 mV/mm stimulation. Green: FITC‐TMEM16A; Red: TRITC‐PDLIM7. Scale bar: 10 µm. Right: Front‐to‐rear intensity ratios. Unpaired Student's *t*‐test (^***^ *P* < 0.001, ^****^ *P* < 0.0001). (B) Cell volume measurements after TMEM16A or SK inhibition under electric field (mean ± SD, *n* ≥ 6). One‐way ANOVA (^*^ *P* < 0.05, ^****^ *P* < 0.0001, ns: not significant). (C) Up: Membrane potential dye images post‐inhibitor treatment. Bottom: Quantification of membrane potential changes at MDA‐MB‐231 cells front and rear. Unpaired Student's *t*‐test (^***^ *P* < 0.001, ns: not significant). Scale bar: 10 µm. (D) Quantification of membrane potential changes (mean ± SD, *n* ≥ 6). Unpaired Student's *t*‐test (^***^ *P* < 0.001, ns: not significant). (E, G) Current traces of TMEM16A and SK channels post‐inhibitor treatment. (F,H) Quantification of TMEM16A and SK current density. (mean ± SD, *n* ≥ 6). Unpaired Student's *t*‐test (^***^ *P* < 0.001). (I,K) Time‐lapse imaging of K⁺ and Cl^−^ under inhibitor treatment (mean ± SD, *n* ≥ 6). Scale bar: 10 µm. (J,L) Quantification of K⁺ and Cl^−^ fluorescence intensity at MDA‐MB‐231 cells front and rear after treatment with control, electric field and inhibitor. (mean ± SEM, *n* ≥ 6 cells). Unpaired Student's *t*‐test (ns: not significant, ^***^ *P* < 0.001, ^****^ *P* < 0.0001). (M) Up: FRET images of Vimentin‐M‐cpstFRET expressing cells post‐inhibitor treatment. Bottom: Normalized CFP/FRET ratios at MDA‐MB‐231 cells front and rear. Unpaired Student's t‐test (^***^ *P* < 0.001, ns: not significant). Scale bar: 10 µm. Calibration bar: 0.00–1.30. (N) Quantified front‐to‐rear tension ratios (mean ± SD, *n* ≥ 6). Unpaired Student's *t*‐test (^***^ *P* < 0.001, ns: not significant).

Immunofluorescence imaging demonstrated that TMEM16A was enriched at the leading edge, whereas SK localized to the trailing edge during electrotactic migration (Figure 6A). Disruption of TMEM16A and SK channels attenuated anodal‐directed cell volume expansion at the leading edge and cathodal‐directed volume reduction at the trailing edge (Figure 6B; Figure S15G). Correspondingly, membrane potential shifts, depolarization at the leading edge and hyperpolarization at the trailing edge were significantly diminished upon channel inhibition (Figure 6C,D). Patch‐clamp recordings further confirmed these observations, revealing reduced membrane potential changes and channel currents in response to histone and albumin stimulation following TMEM16A or SK channel inhibition (Figure 6E–H; Figure S16A,B). Compared to controls, histone and albumin treatments enhanced ion currents through TMEM16A and SK channels, respectively, which were effectively suppressed by the specific inhibitors T16Ainh‐A01 (TMEM16A) and NS8593 (SK). Inhibition of TMEM16A and SK also led to a reduction in Cl^−^ concentrations at the leading edge and K⁺ concentrations at the trailing edge (Figure 6I–L; Figure S15H,I). FRET‐based tension measurements further demonstrated that inhibition of TMEM16A and SK channels significantly reduced Vimentin tension at both the leading and trailing edges (Figure 6M,N). Additionally, immunofluorescence experiment showed that SK activation‐associated PIP2 accumulation was observed at the trailing edge of MDA‐MB‐231 cells (Figure S15J). These findings indicate that TMEM16A and SK channels are key mediators of osmotic pressure regulation at the cell front and rear, respectively, coordinating membrane potential dynamics and tension asymmetry during electrotactic migration.

### 2.6 PKA Polarization is Regulated by TMEM16A‐ and SK‐Mediated Osmotic Pressure, and Dual Knockdown of TMEM16A and SK Inhibits Breast Cancer Cell Electrotactic Migration

**FIGURE 7:**
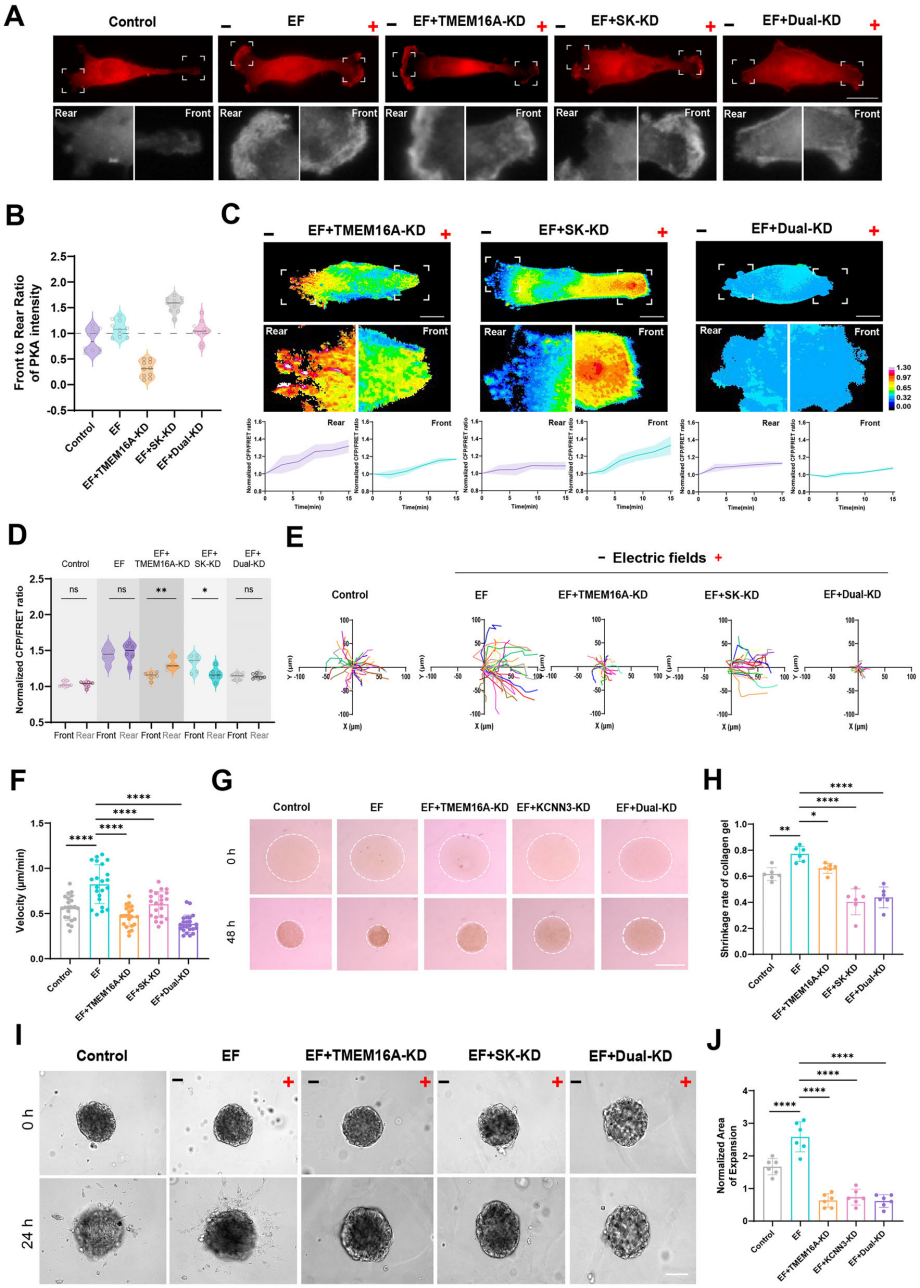
Dual knockdown of TMEM16A and SK impairs electrotactic migration of breast cancer cells. (A) Immunofluorescence images of PKA distribution in TMEM16A‐KD, SK‐KD, and dual‐KD cells under 400 mV/mm stimulation. Scale bar: 10 µm. (B) Quantification of front‐to‐rear PKA intensity ratios (mean ± SEM, *n* ≥ 10 cells). (C) FRET images of PDLIM7‐M‐cpstFRET expressing cells with single or dual knockdown. Scale bar: 10 µm. Calibration bar: 0.00–1.30. (D) Quantified tension ratios (mean ± SD, *n* ≥ 6). Unpaired Student's *t*‐test (^*^ *P* < 0.05, ^**^ *P* < 0.01, ns: not significant). (E) Cell migration trajectories (*n* = 20). (F) Quantification of migration velocity (mean ± SD). One‐way ANOVA (^****^ *P* < 0.0001). (G) Contraction of collagen gels containing knockdown cells at 0 and 48 h. Scale bar: 5 mm. (H) Quantification of gel area (mean ± SD, *n* = 6). One‐way ANOVA (^*^ *P* < 0.05, ^**^ *P* < 0.01, ^****^ *P* < 0.0001). (I) Dissemination of spheroid‐embedded knockdown cells in collagen gels at 0 and 24 h. (J) Quantification of spheroid expansion (mean ± SD, *n* = 6). One‐way ANOVA (^****^ *P* < 0.0001).

Given the critical roles of TMEM16A and SK channels in regulating osmotic pressure asymmetry, we next investigated their functional contributions to breast cancer cell electrotactic migration. Immunofluorescence analysis revealed that individual knockdown of TMEM16A and SK selectively impaired PKA recruitment at the leading and trailing edges, respectively (Figure 7A,B). To assess the impact on cytoskeletal tension, PDLIM7‐M‐cpstFRET probes were transfected into TMEM16A‐knockdown (TMEM16A‐KD), SK‐knockdown (SK‐KD), and dual‐knockdown cells. FRET analysis showed that TMEM16A knockdown significantly reduced PDLIM7 tension at the leading edge, whereas SK knockdown diminished tension at the trailing edge. Dual knockdown, however, resulted in a global reduction of PDLIM7 tension across the entire cell (Figure 7C,D). Functionally, TMEM16A‐KD, SK‐KD, and dual‐KD cells exhibited impaired electrotactic migration compared to control cells. Notably, the migration velocity of dual‐KD cells was significantly reduced, underscoring the cooperative role of these channels in directional cell movement (Figure 7E,F). To evaluate cellular contractility, a gel deformation assay was conducted by quantifying volume changes in collagen gels embedded with cells. TMEM16A‐KD, SK‐KD, and dual‐KD cells exhibited markedly reduced gel deformation compared to control cells, indicating compromised contractile force generation (Figure 7G,H). Furthermore, 3D spheroid assays demonstrated that dual knockdown of TMEM16A and SK was significantly more effective than individual knockdowns in suppressing MDA‐MB‐231 cell dissemination from spheroids (Figure 7I–J). These findings highlight that TMEM16A‐ and SK‐mediated osmotic pressure asymmetry is essential for PKA polarization and cytoskeletal tension during electrotactic migration. Dual inhibition of TMEM16A and SK channels effectively disrupts breast cancer cell migration and invasion, suggesting a promising therapeutic strategy for limiting tumor dissemination and metastasis.

## 3 Discussion

This study investigated the role of asymmetric OP and PDLIM7/microfilament tension in the electrotactic migration of breast cancer cells, elucidating the underlying electromechanical‐synergistic mechanism (Figure S17). We propose that this localized OP distribution governs electromechanical regulation, ultimately determining the migration direction of tumor cells. This finding offers new insights into how electric fields influence tumor cell motility.

Traction protein‐mediated polarization of microfilament tension is critical for cell migration [38, 39]. During anode‐directed migration of MDA‐MB‐231 cells, PDLIM7 tension was markedly increased at both the leading and trailing edges, accompanied by polarized microfilament recruitment. Similarly, other traction proteins, such as srGAP2, talin, and Ezrin, transmit forces from extracellular stimuli to the cytoskeleton, facilitating migration by modulating microfilament network dynamics [7, 40, 41]. PDLIM7 tension polarization is regulated through phosphorylation within its IDR, driven by upstream PKA signaling. Phosphorylation within the IDR alters local charge density [8, 42], such as introducing negative charges at Ser190, potentially modulating phase separation and enhancing PDLIM7's binding to F‐actin. Electric field stimulation specifically aggregates PKA at the leading and trailing edges, reinforcing this phosphorylation effect. These findings highlight the pivotal role of PDLIM7 phosphorylation in coordinating cell migration through kinase polarization and mechanical signal amplification. However, the bilateral recruitment of these traction proteins at both edges suggests that they do not determine the migration direction, raising a key question: which factors dictate the directionality of electric field‐induced migration?

Importantly, our study reveals an innovative role of OP asymmetry in regulating electrotaxis‐driven migration. Osmotic tension generates local pressure gradients within migrating cells [43]. Hyperosmotic expansion at the leading edge and hypoosmotic contraction at the trailing edge collectively drive directional movement. Interestingly, both at the leading and trailing edges are accompanied by the increased PDLIM7 and microfilament tension [22, 44]. Inward‐pulling force through PDLIM7‐microfilament tension and high OP outward‐pushing force, collectively enhance leading‐edge pressure per unit and forming expansion bulge according to a shrinking model [45]. Throughout migration, the osmotic tension gradient between cell front and rear is dynamically regulated by the polarized distribution of ion channels and aquaporins [46, 47, 48]. Chloride ion influx accompanies with high OP, driving water inflow via aquaporins and resulting in local expansion at the cell front [21, 49, 50]. Conversely, potassium efflux following with the low OP promotes the water efflux, leading to volume reduction at the trailing edge [51, 52]. Nuclear deformation also induces enhancement of intermediate filament tension to generate trailing shrinkage. Therefore, it is a vector model of intracellular multiple tensions that control directional migration of tumor cells.

The directionality of electrotaxis‐induced migration is further regulated by an imbalance between membrane depolarization and hyperpolarization [36, 53]. Under electric field stimulation, the leading edge depolarizes while the trailing edge hyperpolarizes as cells migrate toward the anode. Previous studies from our group have shown that intracellular protein nanoparticles (PNs) and calcium ions synergistically influence membrane potential transitions [54]. Electric fields induce PNs accumulation at both cell edges. Negatively charged PNs absorb cations, reorganizing local ion distributions near the membrane. Crucially, localized calcium influx modulates PNs‐induced membrane potential alterations. According to the electrical double‐layer theory, multivalent Ca^2^⁺ ions displace monovalent Na⁺ and K⁺ ions absorbed by PNs, promoting membrane depolarization at the leading edge. Therefore, the PN/Ca^2^⁺ ratio critically determines local membrane potential states. At the leading edge, high Ca^2^⁺ concentrations coupled with lower PNs levels facilitate free monovalent cation inside the cell membrane, driving depolarization. Conversely, an excess of PNs and relatively lower Ca^2^⁺ at the trailing edge leads to adsorption between PNs and monovalent cation, triggering membrane hyperpolarization.

This electric field‐induced membrane potential polarity orchestrates the activation of voltage‐dependent ion channels [55, 56]. The leading edge, facing the anode, recruits chloride channels such as TMEM16A, promoting cellular expansion. At the trailing edge, facing the cathode, SK potassium channels are recruited, facilitating cell shrinkage. The Gardos effect explains how OP‐dependent volume regulation via calcium‐activated potassium channel induces cellular dehydration and contraction [57, 58]. Therefore, the polarized distribution of specific ion channels establishes ionic gradients across the cell, promoting directional migration.

This study establishes the ‘electro‐OP‐tension’ model in breast cancer. Electrotaxis has been documented in glioblastoma and lung adenocarcinoma, where similar electromechanical coupling may underpin directed migration [59, 60]. Meanwhile, these insights carry translational implications: phosphorylated PDLIM7 may serve as a prognostic biomarker for metastatic tumors, enabling targeted surveillance. Therapeutically, pharmacological targeting of PDLIM7 or key ion channels could yield an ‘anti‐metastatic’ strategy that paralyzes dissemination without killing cells. While further validation is needed, this study provides a mechanistic blueprint for targeting electromechanical drivers of metastasis.

In conclusion, we propose a three‐signal polarized integration model, “electro‐OP‐tension,” membrane potential differences between edges and ion channel (TMEM16A/SK) polarization trigger OP oscillations of leading‐edge expansion and trailing‐edge contraction, which subsequently regulate microfilament tension via PKA‐mediated PDLIM7 phosphorylation. This model delineates how electromechanical interactions among membrane potential, ion channels, OP, traction proteins, and cytoskeletal tensions coordinate tumor cell migration. Future studies should aim to develop early‐warning systems for tumor invasion based on OP asymmetry monitoring and further investigate the pathological interplay between electric fields and the tumor microenvironment.

## 4 Experimental Section

### 4.1 Cell Culture

Human MDA‐MB‐231 (RRID: CVCL_0062, Cat: iCell‐h133), MDA‐MB‐468 (RRID: CVCL_0419, Cat: iCell‐h138) and MCF‐7 (RRID: CVCL_0031, Cat: iCell‐h129) cell lines were purchased from Cellverse Co., Ltd on March 11, 2023. The source of the cell lines is human breast cancer. All the above cell lines were identified by STR and were free from mycoplasma contamination. Human MDA‐MB‐231 cells and MDA‐MB‐468 cells were cultured in Leibovitz L‐15 Medium (Gibco) supplemented with 10% heat‐inactivated FBS (Gibco) and 1% penicillin/streptomycin (10,000 U/mL, Gibco). MCF‐7 cells were cultured in Dulbecco's Modified Eagle's Medium (Gibco) supplemented with 10% heat‐inactivated FBS (Gibco) and 1% penicillin/streptomycin (10,000 U/mL, Gibco). Cells were maintained in an incubator at 37 °C with 95% air/5% CO_2_ and passaged upon 60–80% confluency every 3–5 days.

### 4.2 EF Stimulation and Time‐Lapse Image Recording

Direct current electric fields (DCEFs) were applied through agar‐salt bridges connecting with silver/silver chloride electrodes in Steinberg's solution (consisting 58 mm NaCl, 0.67 mm KCl and 0.44 mm Ca (NO_3_)_2_, 1.3 mm MgSO_4_ and 4.6 mm Tris base, pH 7.4) to pooled medium on either side of the galvanotaxis chamber, which was the unidirectional flow of charged particles along the same direction [61]. Cells were exposed to 0, 200 and 400 mV/mm direct current EF for 15 min. Cells were imaged using a Leica SP8 FALCON confocal inverted microscope, equipped with a 63× plan apo (1.4 NA) oil‐immersion objective and serial time‐lapse images were captured. The images were acquired every 1 min. Autonomous moving cells were randomly selected for each experiment, and their trajectories were recorded. Cell migration was analyzed to determine track speed by using ImageJ software (NIH, Bethesda, MA, USA) with MTrackJ and Chemotaxis tool plugins. Briefly, trajectories of cells were pooled to make composite graphs. Speed is the total length travelled by the cells divided by time. Time‐lapse images were analyzed using NIH ImageJ software.

### 4.3 Reagents and Antibodies

Blebbistatin (Bleb) was from Aladdin (Shanghai, China). Y‐27632 (ROCK inhibitor; HY‐10071), 8‐Bromo‐cAMP sodium salt (PKA activator; HY‐12306), H‐89 dihydrochloride (PKA inhibitor; HY‐15979A) and GSK193874 (TRPV4 inhibitor; 1336960‐13‐4) were obtained from Med Chem Express (Monmouth Junction, NJ, USA). Amiodarone (Kir inhibitor; 1951‐25‐3), NS8593 (SK inhibitor; 875755‐24‐1), Paxilline (BK inhibitor; 57186‐25‐1), Senicapoc (IK inhibitor; 289656‐45‐7), 9‐Phenanthrol (TRPM4 inhibitor; 484‐17‐3), T16Ainh‐A01 (TMEM16A inhibitor; 552309‐42‐9), DCPIB (VSOR inhibitor; 82749‐70‐0), CFTRinh‐172 (CFTR inhibitor; 307510‐92‐5), Dooku1 (Piezo1 inhibitor; 2253744‐54‐4), TGN‐020 (AQP4 inhibitor; 51987‐99‐6), EIPA (NHE1 inhibitor; 1154‐25‐2), Sotrastaurin (PKC inhibitor; 425637‐18‐9) and KN‐93 (CaMKII inhibitor; 139298‐40‐1) were obtained from TargetMol (shanghai, China). Gd^3+^ (Max‐Cl inhibitor; W15057‐1g) was obtained from Yuanye (shanghai, China). The following antibodies were used: Rabbit anti‐PDLIM7 (Proteintech, #10221‐1‐AP); Mouse anti‐PDLIM7 (Santa, #sc‐398100); Rabbit anti‐PRKACB (Proteintech, #12232‐1‐AP); Mouse anti‐p‐Ser (Santa, #sc‐81514); Mouse anti‐GFP Tag(6A2) (Zenbio, #250065); Mouse anti‐TMEM16A (Santa, #sc‐377115); Rabbit anti‐KCNN3 (Affinity, #DF13233).

### 4.4 Plasmid Construction and Small Interfering RNA (siRNA) Design

The pCMV3‐PDLIM7‐t1 (HG18621‐UT) was obtained from Sino Biological (Beijing, China). The FRET‐based force probes were constructed using a NovoRec PCR Seamless Cloning Kit (Thermo Fisher Scientific, Waltham, MA, USA) and restriction enzyme cloning. We constructed the PDLIM7 fluorescence sensor with circularly permutated enhanced cyan fluorescent protein (eCFP) and enhanced yellow fluorescent protein (eYFP) [mTurquoise2‐7aa‐super (s) YFP2 circularly permuted stretch‐sensitive FRET (cpstFRET)], which was inserted between aa 278 and aa 279 of PDLIM7. The mutation plasmids (PDLIM7‐S95A, PDLIM7‐S109A, PDLIM7‐S144A, PDLIM7‐S190A and PDLIM7‐S190E) were constructed using KOD‐Plus‐Neo (TOYOBO, Osaka, Japan). All Plasmids were then extracted from individual colonies and purified with Endo‐free Plasmid Mini Kit (OMEGA, USA). The integrity of all expressed structures was confirmed by DNA sequencing. Plasmids encoding PDLIM7 tension sensor were transfected into MDA‐MB‐231 cells with Lipofectamine^TM^ 2000 Transfection Reagent (Invitrogen, Carlsbad, CA, USA) and Opti‐MEM^TM^ media (Invitrogen, Carlsbad, CA, USA) according to manufacturer instructions. The transfection efficiency was about 70%.

The small interfering RNAs (siRNAs) targeting PDLIM7, SLCO2A1, TMEM16A, and KCNN3, and the negative control siRNA (NC) were designed by Genepharma (Shanghai, China) as following:

PDLIM7 sense: 5′‐CAUGCCUUCUCUCAUGUGUTT‐3′

Antisense: 5′‐ACACAUGAGAGAAGGCAUGTT‐3′

SLCO2A1 sense: 5′‐GCUGCAGCAACAUCAACAUTT‐3′

Antisense: 5′‐AUGUUGAUGUUGCUGCAGCTT‐3′

TMEM16A sense: 5′‐GAGGAGUCAACAAACAAAUTT‐3′

Antisense: 5′‐AUUUGUUUGUUGACUCCUCTT‐3′

KCNN3 sense: 5′‐CGGGAAACAUGGUUAAUCUTT‐3′

Antisense: 5′‐AGAUUAACCAUGUUUCCCGTT‐3′

NC sense: 5′‐UUCUCCGAACGUGUCACGUTT‐3′

Antisense: 5′‐ ACGUGACACGUUCGGAGAATT‐3′

### 4.5 Kaplan–Meier Survival Analysis

Analysis for breast cancer metastasis was performed using Kaplan–Meier plotter (https://kmplot.com). Auto scan mode was utilized to choose the cutoff between high and low expression cohorts.

### 4.6 Transwell Assay

The upper chamber of 24‐well transwell plates (pore size: 8 µm; Corning, NY, USA) was precoated with 50 µL of matrigel solution. MDA‐MB‐231 cells (2×10^5^) were starved overnight and seeded into the upper chamber in serum‐free medium, and medium supplemented with 20% FBS was added to the bottom chamber. After 24 h incubation, the cells were fixed with 4% paraformaldehyde and dyed with crystal violet for 15 min. Typical images of invading cells were obtained for statistical analyses. Images were captured for each group at least three fields and three independent experiments. The migration assay was performed in a similar manner to the invasion assay but without the Matrigel pre‐coating. Cells were imaged using DIC model of Lecia DMI8 microscope with a 10× objective lens under constant exposure (100 ms) and gain (1.0).

### 4.7 Immunofluorescence Analysis

For immunostaining with PDLIM7, PRKACB, TMEM16A, or KCNN3 antibodies, cells were fixed with 4% formaldehyde solution (ThermoFisher Scientific), permeabilized with 0.1% Triton X‐100 (Sigma–Aldrich), blocked with 5% BSA, immunostained, and imaged with a Leica SP8 inverted fluorescence microscope (Leica, Wetzlar, Germany) equipped with a 63x plan apo (1.4 NA) oil‐immersion objective. Primary antibodies were used at the following concentrations: Rabbit anti‐PDLIM7 (1:100, Proteintech, #10221‐1‐AP); Mouse anti‐PDLIM7 (1:50, Santa, #sc‐398100); Rabbit anti‐PRKACB (1:100, Proteintech, #12232‐1‐AP); Mouse anti‐TMEM16A (1:50, Santa, #sc‐377115); Rabbit anti‐KCNN3 (1:100, Affinity, #DF13233). Following overnight incubation with primary antibodies at 4°C, specimens were washed with PBS, and then secondary antibodies were applied for 2 h at room temperature: Alexa Fluor 488 goat anti‐mouse immunoglobulin‐G (IgG) (H + L) (1:100, ZF‐0313), Alexa Fluor 488 goat anti‐rabbit IgG (H + L) (1:200, ZF‐0311), Alexa Fluor Plus 546 goat anti‐mouse IgG (H + L) (1:100, ZF‐0312), or Alexa Fluor Plus 546 goat anti‐rabbit IgG (H + L) (1:100, ZF‐0316) were obtained from Zsgb‐Bio (Beijing, China). Nuclei were stained with DAPI (1:1000, Beyotime C1005). Microfilaments were stained with Phalloidin (1:200, YEASEN, #40735ES75).

### 4.8 Western Blotting

Cells were lysed in radioimmunoprecipitation assay (RIPA) lysis buffer (Beyotime Biotechnology) for 20 min on ice. Samples were pelleted at 12,000×G for 10 min at 4 °C. Lysates were diluted in SDS sample buffer (0.2 M Tris‐HCl, 3% SDS, 10% glycerol, 3% 2‐mercaptoethanol, 0.25% bromophenol blue) and heated at 95°C for 5 min. Lysates were analyzed by electrophoresis on 12% SDS‐PAGE gels at 120 V for 1 h in Tris‐Glycine‐SDS running buffer. Separated proteins were transferred to methanol‐activated PVDF membranes using Turboblot transfer (BioRad). Membranes were incubated in 5% non‐fat milk and blocked for 1 h. Primary antibodies (Rabbit anti‐PDLIM7 (1:1000, Proteintech, #10221‐1‐AP); Mouse anti‐p‐Ser (1:500, Santa, #sc‐81514); Mouse anti‐GFP Tag(6A2) (1:1000, Zenbio, #250065); Mouse anti‐TMEM16A (1:500, Santa, #sc‐377115); Rabbit anti‐KCNN3 (1:500, Affinity, #DF13233); Mouse anti‐GAPDH (1:1000, Zenbio, #380626) were diluted in Li‐Cor buffer at 1:500 dilution and incubated overnight at 4°C. Membranes were washed 3x in PBS + 0.1% Tween20 (TBST). Membranes were incubated with secondary anti‐bodies [anti‐mouse IgG HRP‐linked antibody (Zsgb‐Bio) or anti‐rabbit IgG HRP‐linked antibody (Zsgb‐Bio)] at 1:1000 at room temperature for 2 h, washed in TBST and imaged using Li‐Cor CLx. GAPDH was set as control. The enhanced chemiluminescence (ECL) chromogenic substrate was used to visualize the immunoreactive protein bands, and the protein band intensities were quantified using densitometry (Quantity One; Bio‐Rad, Hercules, CA, USA).

For co‐IP experiments, cells were lysed in 0.5% NP‐40 lysis buffer (50 mM Tris‐HCl, pH 8.0, 150 mM NaCl, 0.5% NP‐40, 1 mM DTT) with protease inhibitors cocktail (Selleck). After centrifugation at 16,200 g for 15 min, the supernatants were collected and incubated with primary antibody [Rabbit anti‐PRKACB (3 µg peer100‐500 µg of total protein, Proteintech, #12232‐1‐AP); Rabbit anti‐PDLIM7 (3 µg peer100‐500 µg of total protein, Proteintech, #12221‐1‐AP)] at 4 °C for 8 h followed by incubating with Protein‐A beads (Millipore) for another 4 h at 4 °C. After incubation, samples were washed with lysis buffer for five times. 2 ˣ loading buffer was added to the sample and heated at 95 °C for 5 min. After centrifugation, upper samples were collected. IgG was set as control. Western blots were performed as previously described.

### 4.9 Construction and Testing of the PDLIM7 Tension Probe and FRET Analysis

To detect real‐time changes in PDLIM7 tension, we designed a FRET‐based tension probe. The FRET module was incorporated within the PDLIM7 backbone to report the real‐time resonant energy transfer during angle twisting of the donor‐acceptor pair induced by tension loading onto PDLIM7. FRET acceptor photobleaching (FRET‐AB) test was used to verify the FRET efficiency of PDLIM7 tension probe. We bleached the acceptor with a 514 nm laser at 100% 20 times, and then the fluorescence intensity of the donor and acceptor after bleaching was recorded. The acceptor fluorescence (eYFP) was decreased dramatically upon acceptor photobleaching (AB). Meanwhile, the donor fluorescence (eCFP) was increased due to the unacceptable energy transfer from donor to acceptor after photobleaching. This experiment showed the efficiency of FRET events between the two fluorophores. In the fluorescence recovery after photobleaching (FRAP) test, we selected a region of interest (ROI) and bleached it with a 590 nm laser at 100%. We acquired time‐series images before and after bleaching in 200 s, then recorded the fluorescence intensity in the ROI and calculated the fluorescence recovery rates for PDLIM7 in region of interest (ROI).

For the signal cell FRET analysis, FRET/Acceptor emission ratios were calculated for each pixel clearest optical plane for each image field. The cell was first selected by generating a binary mask using the drawing tool in Image J. A Donor mask was generated by applying a threshold on the Donor image, which was then made binary by converting pixel intensity values greater than the Donor threshold to 1 and those lower than it to 0. A similar mask was generated for the acceptor channel. Ratio images (32‐bit) were calculated the CFP/FRET ratio (the intensity of the CFP channel divided by the intensity of the FRET channel) using the equation E = eCFPdonor/eYFPacceptor, which correlated negatively with the FRET efficiency, but positively with force. For presentation purposes, pseudocolour was applied using Image J software in order to obtain the final images.

### 4.10 Spheroid Formation, and 3D Collagen Invasion Assay

Matrigel was diluted with DMEM containing 10% heat‐inactivated FBS and 1% penicillin/streptomycin at 1:3 ratio. Fifty microliters of the diluted Matrigel was transferred to a 96‐well plate (Falcon) and polymerized for 1 h at 37 °C in a cell culture incubator, while the rest of the diluted Matrigel was kept on ice. MDA‐MB‐231 cells (2×10^5^) were suspended in 50 µL ice‐cold Matrigel and gently plated in different wells pre‐coated with polymerized Matrigel followed by incubation at 37 °C and 5% CO_2_ in a cell culture incubator. After 1.5 h, 100 µL of prewarmed DMEM containing 10% FBS and 1% penicillin/streptomycin was added to each well. The cell culture medium was replaced every two days, and spheroids were used in invasion assays ∼10‐15 days later.

3D collagen invasion assays using spheroids were performed as previously described [62]. Briefly, 3 mL of rat tail collagen type I (Corning) was gently mixed with 375 µL of 10 x DMEM‐low glucose (Sigma). After 1 h incubation on ice, 25 µL of the mixture were added to a 24‐well plate (Falcon) and incubated at 37 °C for 1 h. Spheroids were collected into 1.5 ml Eppendorf tubes by gently disrupting the Matrigel with ice‐cold DMEM. The Eppendorf tube was incubated in ice to further depolymerize the Matrigel for >10 min. Spheroids were isolated by 2665 × g centrifugations for 5 min, and resuspended into 100 µL of the collagen mixture. Next, 100 µL of the spheroid‐collagen mixture were plated in each well and incubated at 37 °C for 1‐1.5 h. After collagen polymerization, 500 µL prewarmed cell culture media was added to each well.

Time‐lapse images were recorded in 20 min intervals for ∼30 h in the IncuCyte Zoom time‐lapse microscopy system (Essen, USA), equipped with a stage‐top incubator at 37 °C and 95% air/5% CO_2_ and an IncuCyte Zoom 10×objective lens. Normalized area expansion and circularity were determined using Image J by outlining the spheroid at t = 0 and 24h using polygonal regions of interest.

### 4.11 Molecular Docking of PDLIM7 to PKA

Molecular docking was used to analyze the intermolecular interactions between PKA and PDLIM7 by predicting binding modes and affinities. PKA were designated as receptors, whereas PDLIM7 served as the ligand for docking validation. The SMILES structure of PDLIM7 and PKA were retrieved from the PubChem database, and the Protein Data Bank (PDB) file was downloaded using its UniProt ID from the PDB database. PyMol was used to preprocess PKA and PDLIM7, including energy optimization, hydrogen addition, water molecule removal, and energy minimization. The docking simulation was carried out using AlphaFold2, and the conformations of the output were sorted according to the binding free energy. The docking site with the lowest score was selected as the optimal binding mode. Binding energies were calculated, and the binding mode was visualized in 3D. Corresponding 3D docking images were generated in PyMol to illustrate hydrogen bonds, hydrophobic interactions, and other key interactions.

### 4.12 Cell Volume Measurements

The MDA‐MB‐231 cells were cultured in confocal 35 mm petri dishes and the density was about 30%. Cell volume was calculated using a fully automated 360‐degree labeling free live cell 3D imaging system which equipped with 60 x objective. The cells were holographic scanned by a 360‐degree rotating light source, holographic 3D imaging based on the cell's physical refractive index RI. STEVE Software (Nanolive CXA, Trochner, Switzerland) was applied to analyze changes in cell volume.

### 4.13 Patch‐Clamp Experiments

Whole‐cell recordings were obtained using a Multiclamp 700B amplifier and a Digidata 1550B digitizer (Molecular Devices) controlled by pClamp10.6 software (Molecular Devices, Sunnyvale, CT, USA). Pipettes were made of borosilicate glass (O.D.: 1.5 mm from Sutter Instruments) and fire‐polished to obtain a resistance between 2–5 MΩ. All experiments were conducted at room temperature (23‐25°C). Capacitance compensation and series resistance compensation were adjusted before recording. Data was acquired at 10 kHz and filtered at 2 kHz. The pClamp10.6 software (v.10; Axon Instruments) was used for pulse generation, data acquisition, and subsequent analysis.

The external solution contained (in mM): NaCl 90, KCl 4.8, MgCl_2_ 2.4, CaCl_2_ 2.5, NaHCO_3_ 5 and HEPES 10 (pH 7.4), osmotic pressure 300 mOsm/kg. The pipette solution contained (in mM): KCl 139, MgCl_2_ 2, Na_2_ATP 2, CaCl_2_ 1, EGTA 2 and Glucose 5 (pH 7.4), osmotic pressure 300 mOsm/kg. Immediately after the whole cell configuration, current clamp mode was used to measure resting membrane potential (RMP), input resistance (Rin), and albumin (1mg/mL) and histone (0.1mg/mL) stimulated. TMEM16A‐like chloride currents in MDA‐MB‐231 cells were measured clamped at 0 mV and pulsed for 300 ms from ‐100 mV to +100 mV in 20 mV steps [63]. T16Ainh‐A01 (MCE, # 552309‐42‐9) was dissolved in DMSO and diluted with external solution, which was used to inhibit TMEM16A channels. KCNN3‐like chloride currents in MDA‐MB‐231 cells were measured clamped at ‐70 mV and pulsed for 300 ms from ‐100 mV to +80 mV in 20 mV steps [64]. NS8593 (MCE, # 875755‐24‐1) was dissolved in DMSO and diluted with external solution, which was used to inhibit KCNN3 channels. All patch‐clamp data were analyzed off‐line using Clampfit v10.6 (Molecular Devices).

### 4.14 Stable Cell Lines and Xenograft Mouse Model

MDA‐MB‐231 cells expressing GFP‐tagged empty vector, GFP‐PDLIM7‐S95A, GFP‐PDLIM7‐S190A, GFP‐PDLIM7‐S190E and GFP‐PDLIM7‐S144A were cultured in medium containing 1000 mg/mL geneticin to screen transfected cells for 7 days. If >90% of cells exhibited fluorescence, subsequent experiments were performed. Thirty 6–8 week‐old female BALB/c nude mice (16‐18 g) were obtained from CAVENS on January 18, 2024 and maintained under specific non‐pathogenic conditions at Nanjing University of Chinese Medicine (Animal License: 202508A028). Animal studies were performed according to the institutional guidelines approved by Institutional Animal Care and Use Committee of Nanjing University of Chinese Medicine. The animals were randomly divided into groups: wild‐type (WT), PDLIM7 S190A, PDLIM7 S190E and PDLIM7 S144A. Each group was injected in situ with MDA‐MB‐231 cells (1×10^6^) suspended in 20 µL Matrigel. Lung nodules were monitored and quantified using Micro CT imaging system (PerkinElmer Company, America) at different time points. On the 28th day, the mice were sacrificed and their organs were immediately removed to obtain evidence of metastatic signals. The lungs of these mice were stained with hematoxylin and eosin.

### 4.15 In Vivo Fluorescence Imaging

GFP‐labeled cells were injected subcutaneously into mice. At the indicated timepoints following injection, mice were anesthetized with 2% isoflurane gas and imaged for the maximum photon emission. Total photon flux (photons/sec) was calculated and corrected for tissue depth by spectral imaging using IVIS Spectrum (PerkinElmer Company, America). Percent primary tumor growth was determined by subtracting the background from the peak signal during each measurement and normalizing it to the initial reading obtained for the same mouse. To avoid false positive detection of bioluminescence signal, the background subtracted value was normalized to the background reading, and only readings that were ≥50x higher than the background reading were considered to be positive for metastasis.

### 4.16 Immunohistochemistry and Pathology

Animals with primary tumors exceeding the endpoint (e.g., signal saturation >1000‐fold above initial) were sacrificed. Sacrificed mice underwent immediate organ removal to obtain evidence of metastatic signals. Tissue samples were removed, fixed in formalin for 24 h, embedded in paraffin wax, and serially sectioned (4 µm thick). The lungs of these mice were stained with hematoxylin and eosin, and the lung nodules in serial sections were microscopically quantified.

### 4.17 Intracellular Ca^2+^, Cl^−^, K^+^ Ion and Membrane Potential Fluorescence Imaging

Cells were washed with HBSS++ three times. Ca^2+^ ion imaging: the cell‐loading solution consisted of 4 µM Fluo‐4 AM (Beyotime, #S1060) supplemented with Pluronic F‐127 (0.02% v/v) diluted in HBSS++. Excitation and emission were recorded at 494 nm and 516 nm, respectively. Cl^−^ ion imaging: the cell‐loading solution consisted of 10 µM MQAE (TargetMol, #162558‐52‐3) in HBSS++. Excitation and emission were recorded at 350 nm and 460 nm, respectively. K^+^ ion imaging: the cell‐loading solution consisted of 2 µM EPG‐4 AM (MK, #MX4521) in HBSS++. Excitation and emission were recorded at 525 nm and 545 nm, respectively.

Membrane potential fluorescence imaging: Cells were stained with 0.1 µL M09 fluorescent dye diluted in 100 µL Hank's buffer. The staining solution was added to confocal dishes to fully cover the cells and incubated in the dark for 30 min. Fluorescence was recorded at excitation and emission wavelengths of 490 nm and 516 nm, respectively. An increase in fluorescence intensity indicates cellular depolarization, leading to an influx of the probe, whereas a decrease signifies hyperpolarization and subsequent efflux of the probe. To validate the dye's positive responses, cells were depolarized with an equal volume of buffered, isotonic potassium chloride (KCl) solution. Typical dye responses approached 25% dF/F per 100 mV [65]. Cells were incubated in the cell‐loading solution at room temperature for 45 min. After incubation, cells were washed with HBSS++ three times. To allow for AM‐deesterification, cells were incubated for another 30 min in HBSS++ at room temperature.

Confocal microdish was placed into the Attofluor cell chamber (Thermo Fisher Scientific) and imaged using a Leica SP8 inverted fluorescence microscope (Leica, Wetzlar, Germany) under a 20 ×/0.75 NA objective. Images were taken every 3 min, and EF (400 mV/mm) was added after 60 s of baseline measurement. Image J software (National Institutes of Health) was applied to quantified fluorescence intensity. Relative fluorescence intensities were expressed as F_t_/F_0_, where F_t_ was the fluorescence intensity and F_0_ was the mean basal fluorescence intensity. Data was plotted with time on the x axis and F_t_/F_0_ on the y‐axis.

### 4.18 Reverse transcription Quantitative Real‐Time Polymerase Chain Reaction (RT‐qPCR)

Total RNA was extracted from MDA‐MB‐231 cells using FreeZol reagent (Vazyme, R711‐01), and was reversely transcribed into cDNA using HiScript II Q RT SuperMix for qPCR (Vazyme, R222‐01) according to the protocols. Then, RT‐qPCR was performed using the ChamQ Universal SYBR qPCR Master Mix (Vazyme, Q711‐02). ACTB (beta actin) was used as an internal control. SLCO2A1 forward primer sequence: 5′‐GCCCATAGGAGCAAAGAGGG‐3′, reverse primer sequence: 5′‐ CCAGGGAGCCTCTTGACTTG‐3′.

### 4.19 Contractility Assay

MDA‐MB‐231 cells (1×10^5^) were harvested and resuspended in desired medium. 2% collagen lattice was prepared to resuspend the cells by mixing 10×PBS, 0.23% 1N NaOH and H_2_O in a tube on the ice, then collagen was added into the tube and mixed intensively. The mixture of cells and collagen were then seeded into a 48‐well plate and cultured at 37°C with 5% CO_2_ for 30 min. The collagen gels were isolated from the well by a pipette and then an appropriate volume of culture medium was added into the wells. Photos of the collagen gels were taken at 0 h and 48 h, and Image J was used to count the area of collagen gels at each time point, and statistical graphs were generated in Graphpad Prism 9.4.

### 4.20 Data Statistics

All data were presented as mean ± standard error of the mean (SEM) or mean ± standard deviation (SD). Datasets exhibiting Gaussian distribution were analyzed using two‐tailed Student's t‐test or one‐way analysis of variance (ANOVA) followed by Tukey's post hoc test. Analysis was performed using SPSS v.22.0 (IBM, Armonk, NY, USA). Specific statistical methods are detailed in the corresponding figure legends. Statistical significance was indicated as follows: not significant (ns), ^*^ *P* < 0.05, ^**^ *P* < 0.01, ^***^ *P* < 0.001, ^****^ *P* < 0.0001.

## Author Contributions

J.G. and H.W. designed the study. L.Z designed and performed the experiments, collected and analyzed the data. Z.Z., H.Z. and Y.H. gave experimental technical support. W.L. performed patch clamp experiment. C.S. performed bioinformatic analysis. L.Z., Y.Z. and X.S. performed the animal experiments and collected the data. L.Z. and Z.Z. drafted the manuscript. All authors approved the final version of the manuscript for submission.

## Conflicts of Interest

The authors declare no conflict of interest.

## Supporting information




**Supporting File**: advs73485‐sup‐0001‐SuppMat.docx.

## Data Availability

The data that support the findings of this study are available from the corresponding author upon reasonable request.
