# Supplementary material for: Osmotic Tension Asymmetry Drives Electrotactic Migration via PDLIM7‐Polarized Microfilament Coordination in Breast Cancer Cells
Source: Adv Sci (Weinh). 2025 Dec 22;13(13):e15246. doi: 10.1002/advs.202515246 (PMC12955868; doi:10.1002/advs.202515246)
Supplement: Supplementary file 1 — Supporting File: advs73485‐sup‐0001‐SuppMat.docx. [file ADVS-13-e15246-s001.docx]

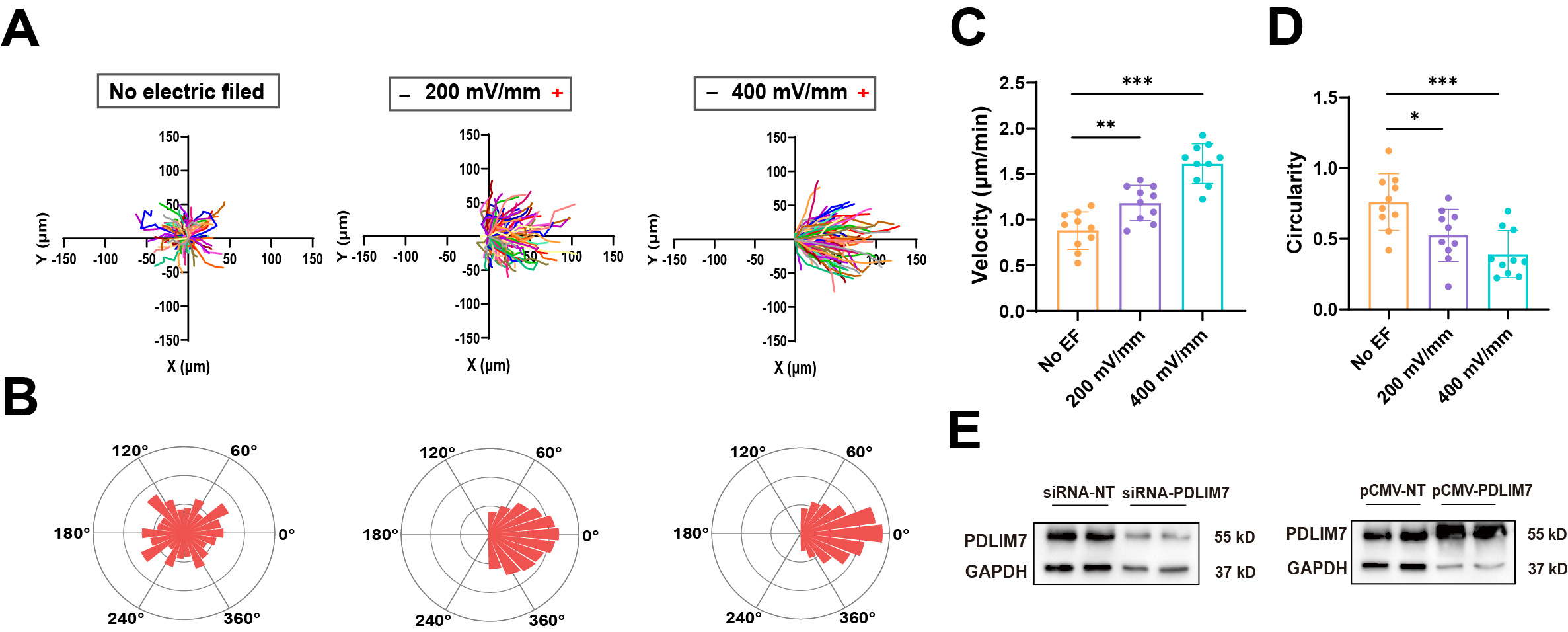
**Support information**

Figure S1. Ex vivo electrotaxis setup and polarity responses of MDA-MB-231 cells.

(A) Migration tracks of MDA-MB-231 cells under no current, low current (200 mV/mm), and high current (400 mV/mm) stimulation for 1 hour (n ≥ 80 cells). (B) Rose plots depicting cell orientation angles; bar lengths represent the fraction of cells within each angular trajectory bin. (C) Cell migration velocity under varying currents (mean ± SD). One-way ANOVA (***P* < 0.01, ****P* < 0.001). (D) Quantification of cell circularity under different electric field conditions (mean ± SD). One-way ANOVA (**P* < 0.05, ****P* < 0.001). (E) Immunoblot analysis of PDLIM7 expression in cells transfected with pCMV-PDLIM7 plasmid or PDLIM7-siRNA.


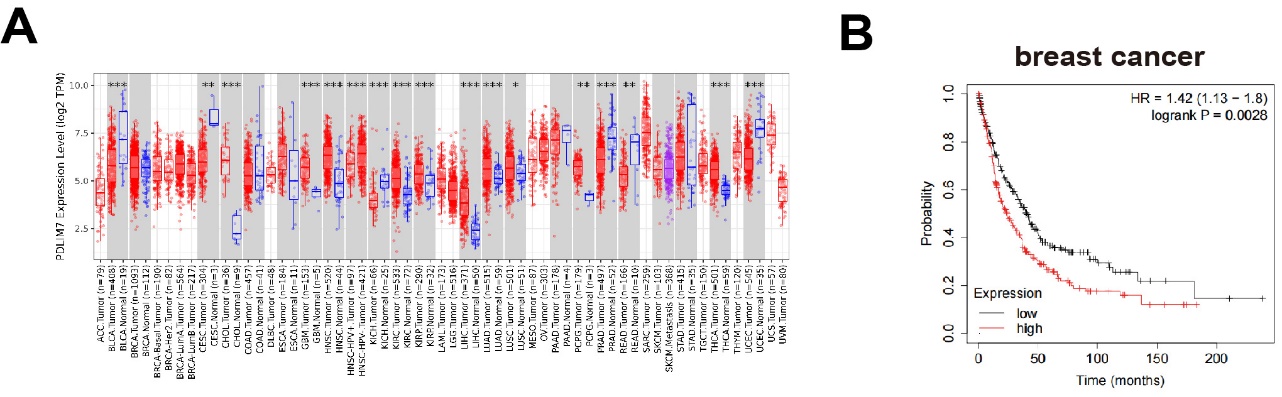
Figure S2. Correlation between PDLIM7 expression and tumor malignancy.

(A) PDLIM7 expression levels in tumor versus normal tissues from The Cancer Genome Atlas (TCGA). (B) Kaplan-Meier survival curves showing overall survival of breast cancer patients stratified by PDLIM7 expression.


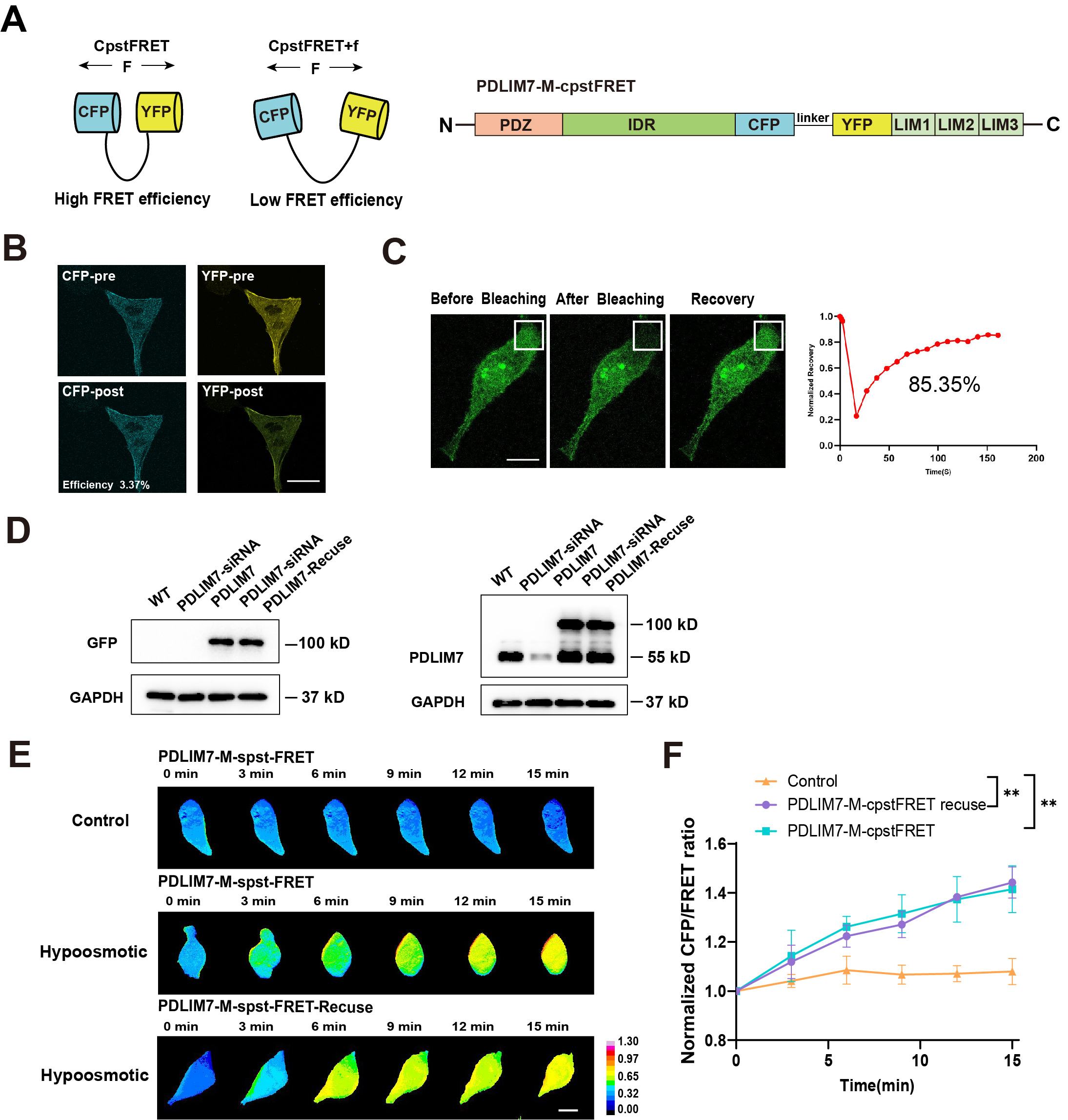
Figure S3. Construction and validation of the PDLIM7 tension probe.

(A) Schematic of PDLIM7 mutation probes. (B) Validation of the PDLIM7-M-cpstFRET probe using acceptor bleaching FRET and FRAP assays. (C) Representative recovery image of PDLIM7-M-cpstFRET after photobleaching; white box indicates bleached area. Quantification of normalized fluorescence recovery over time (n = 6). Scale bar: 10 μm. (D) Immunoblot analysis of PDLIM7 in cells transfected with PDLIM7-M-cpstFRET or PDLIM7-siRNA. (E-F) FRET images and quantification of CFP/FRET ratios after hypotonic stimulation (mean ± SD, n = 6). One-way ANOVA (***P* < 0.01).


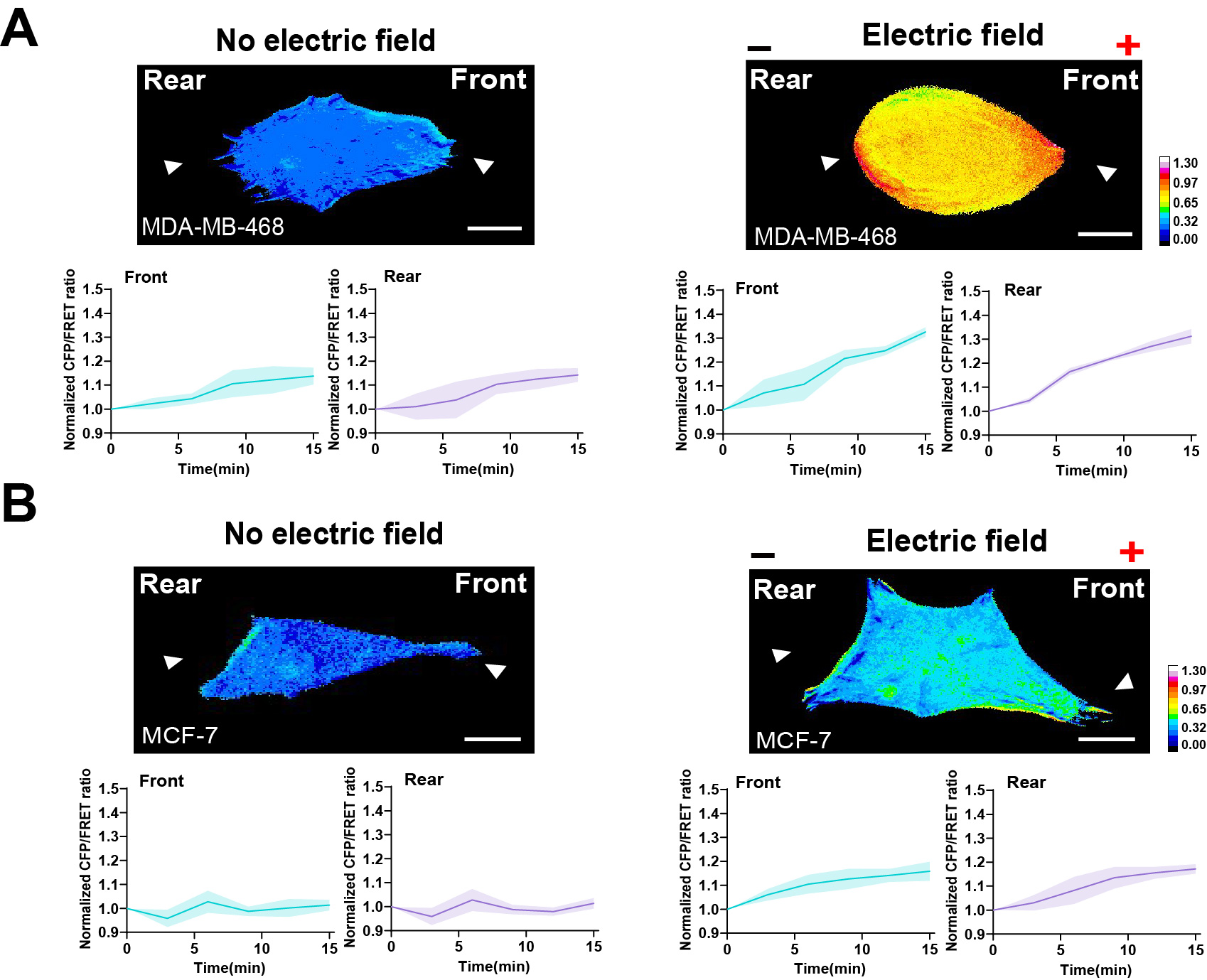
Figure S4. PDLIM7 tension of MDA-MB-468 and MCF-7 cells in electric filed.

(A-B) FRET images and CFP/FRET quantification in MDA-MB-468 and MCF-7 cells under electric field stimulation (mean ± SD, n = 6). Scale bar: 10 μm.


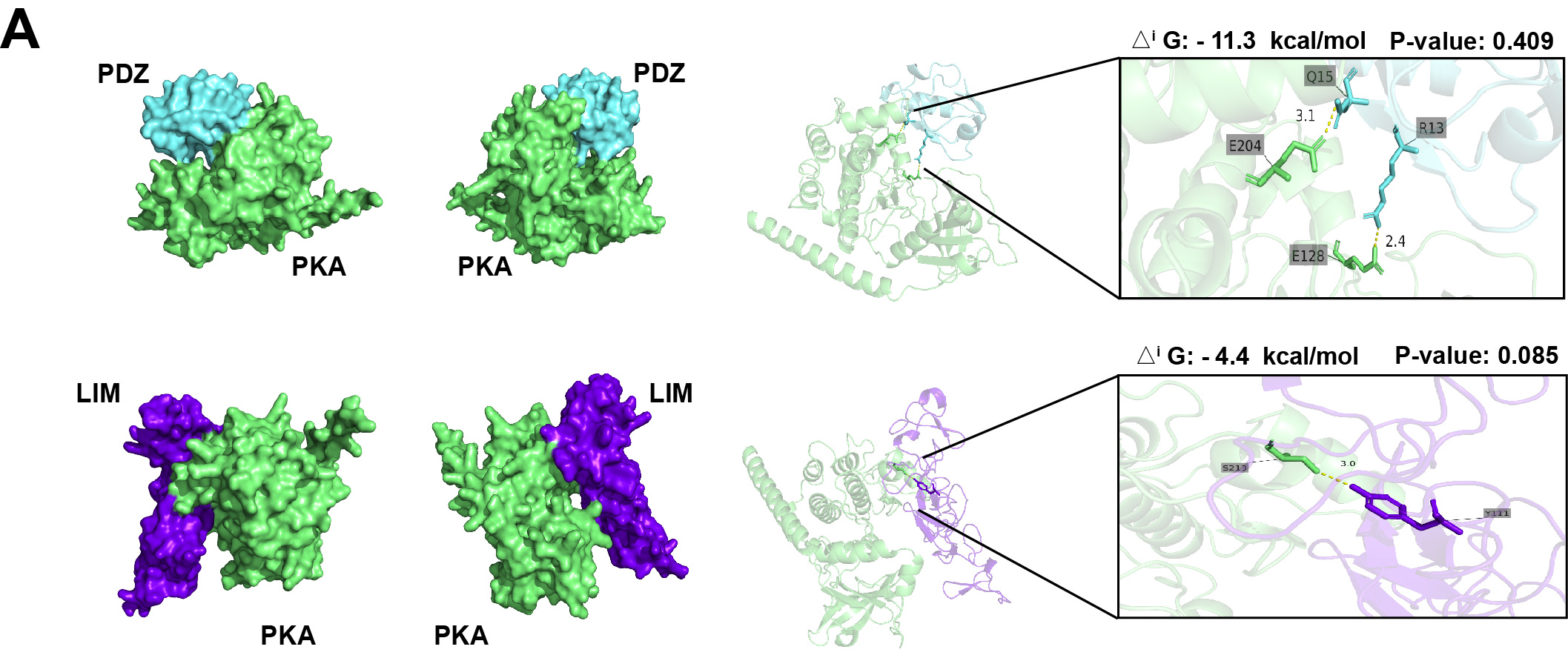
Figure S5. Structural prediction of PDLIM7-PKA interaction.

(A) AlphaFold2-predicted structure of PDLIM7 binding to PKA. Blue: PDZ domain; Purple: LIM domain; Green: PKA. Right: Enlarged view of the binding interface and affinity values.


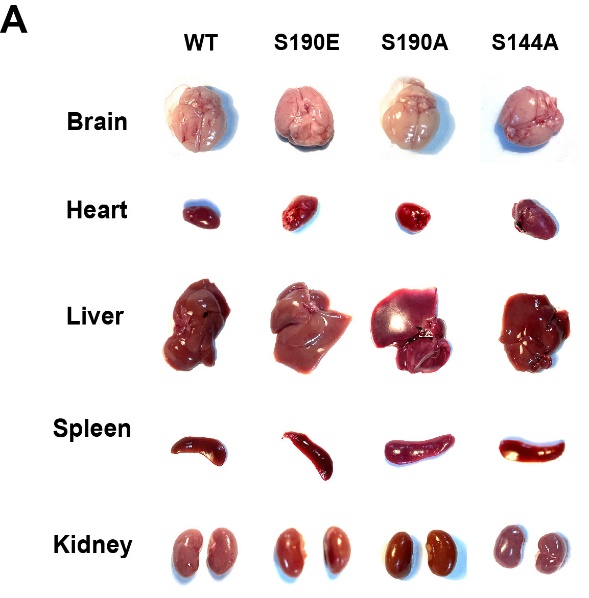


Figure S6. Representative images of organs from nude mice in orthotopic implantation assay.

(A) Gross images of lung, brain, liver, intestine, heart, and pancreas tissues collected from tumor-bearing mice.


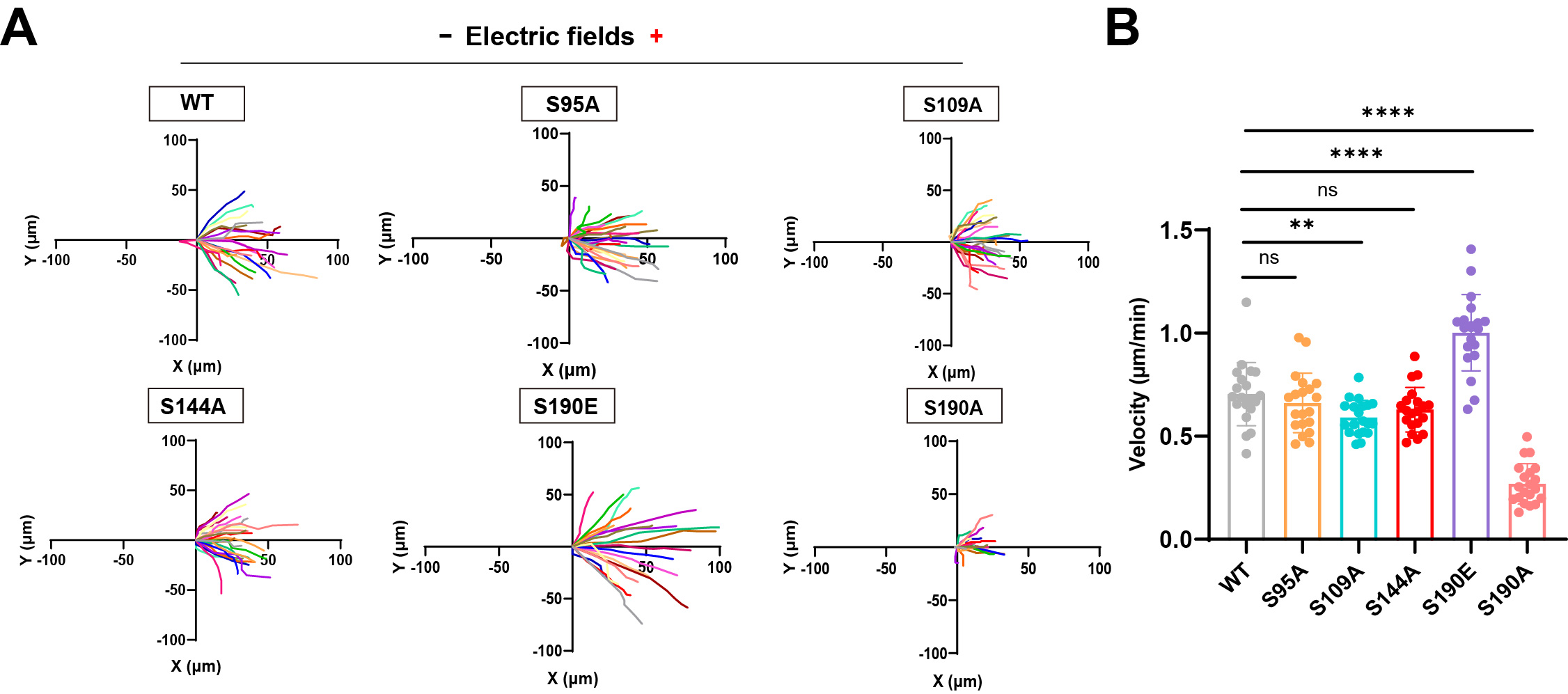


Figure S7. PDLIM7 Ser190 phosphorylation impairs electrotactic migration.

(A) Cell migration trajectories (n≥20). (B) Quantification of migration velocity (mean ± SD). One-way ANOVA (***P* < 0.01, *****P* < 0.0001, ns: not significant).


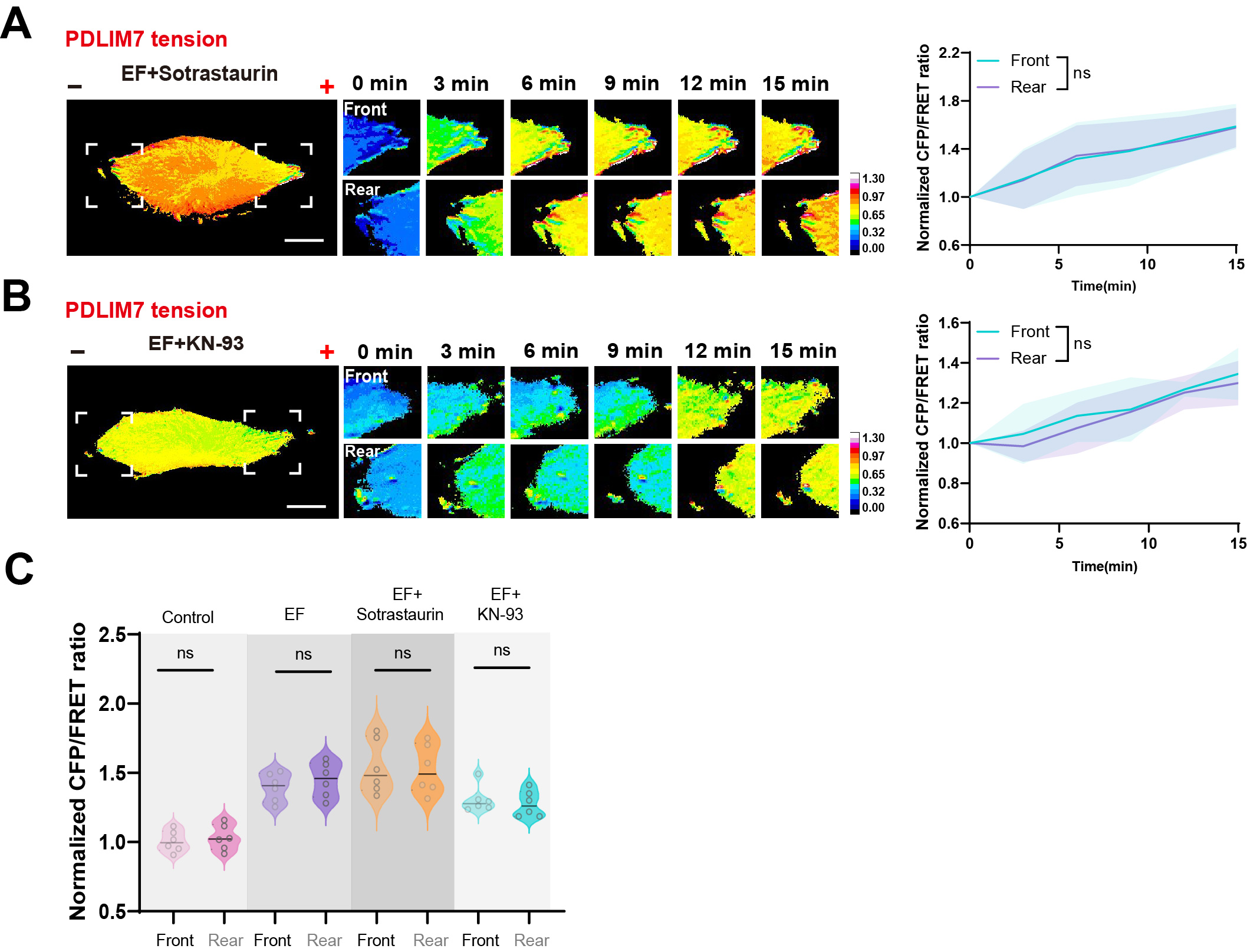


Figure S8. Out of regulation of PDLIM7-mediated tension by PKC or CaMKII.

(A-B) Left, FRET images of PDLIM7-M-cpstFRET expressing cells post-inhibitor treatment. Right, Normalized CFP/FRET ratios at MDA-MB-231 cells front and rear. Unpaired Student’s t-test (ns: not significant). PKC inhibitor: Sotrastaurin (10 µM). CaMKII inhibitor: KN-93 (10 µM). Scale bar: 10 μm. Calibration bar: 0.00-1.30. (C) Quantified front and rear tension CFP/FRET ratios (mean ± SD, n ≥ 6). Unpaired Student’s t-test (ns: not significant).


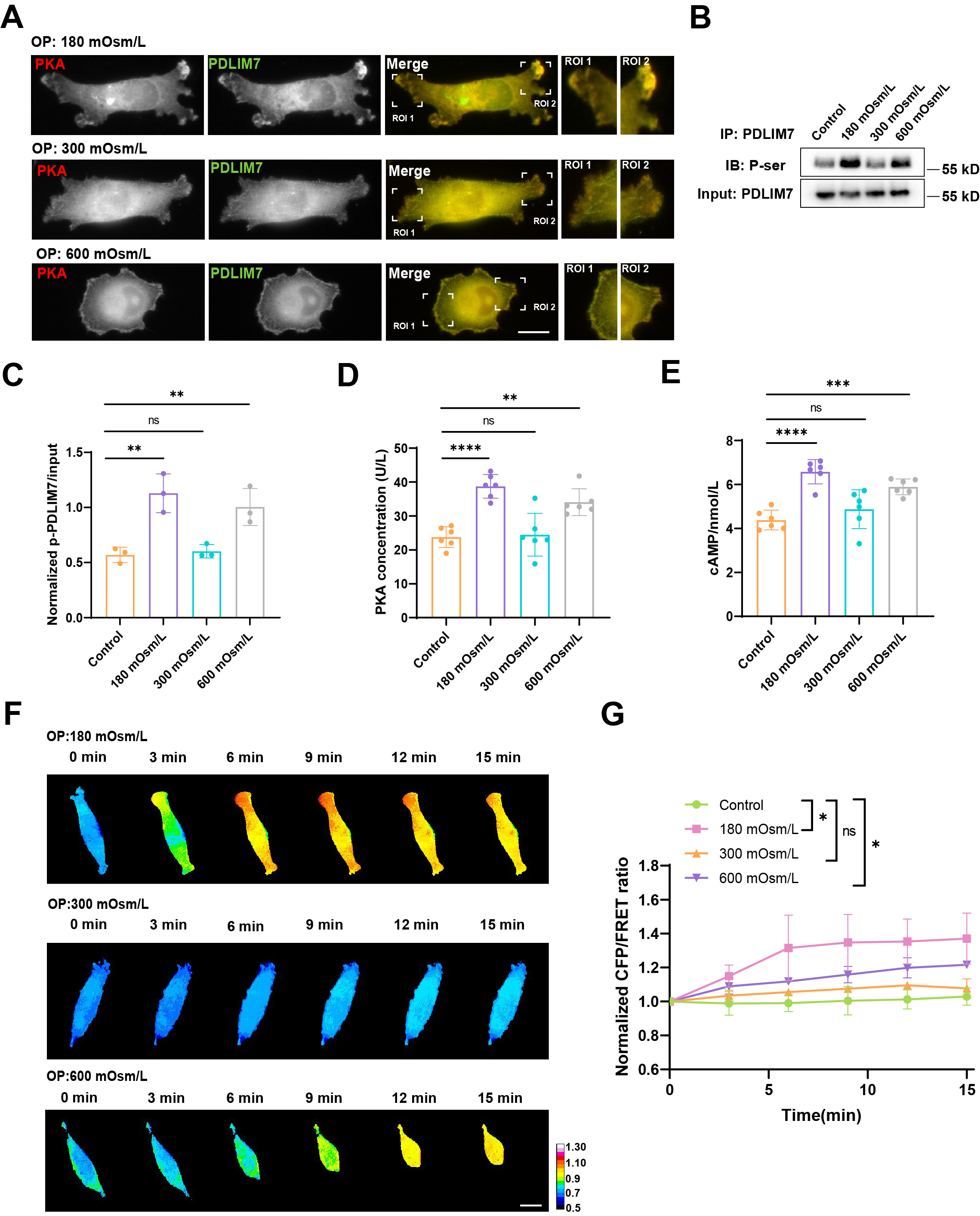


Figure S9. Osmotic pressure regulates PKA recruitment at the cell edge.

(A) Immunofluorescence of endogenous PKA and PDLIM7 under hypoosmotic (180 mOsm/L), isotonic (300 mOsm/L), and hyperosmotic (600 mOsm/L) conditions. Green: FITC-labeled PDLIM7; Red: TRITC-labeled PKA. Scale bar: 10 μm. (B) Immunoprecipitation analysis of phosphorylated PDLIM7 levels under different osmotic pressures. (C) Quantification of phosphorylated PDLIM7/input ratio (n = 3). One-way ANOVA (**P < 0.01, ns: not significant). (D-E) PKA activity and cAMP concentration measurements after osmotic stimulation (mean ± SD, n = 6). One-way ANOVA (**P < 0.01, ***P < 0.001, ****P < 0.0001, ns: not significant). (F-G) FRET images and CFP/FRET ratio quantification in cells expressing PDLIM7-M-cpstFRET under osmotic stimulation (mean ± SD, n ≥ 6). One-way ANOVA (*P < 0.05, ns: not significant). Scale bar: 10 μm.


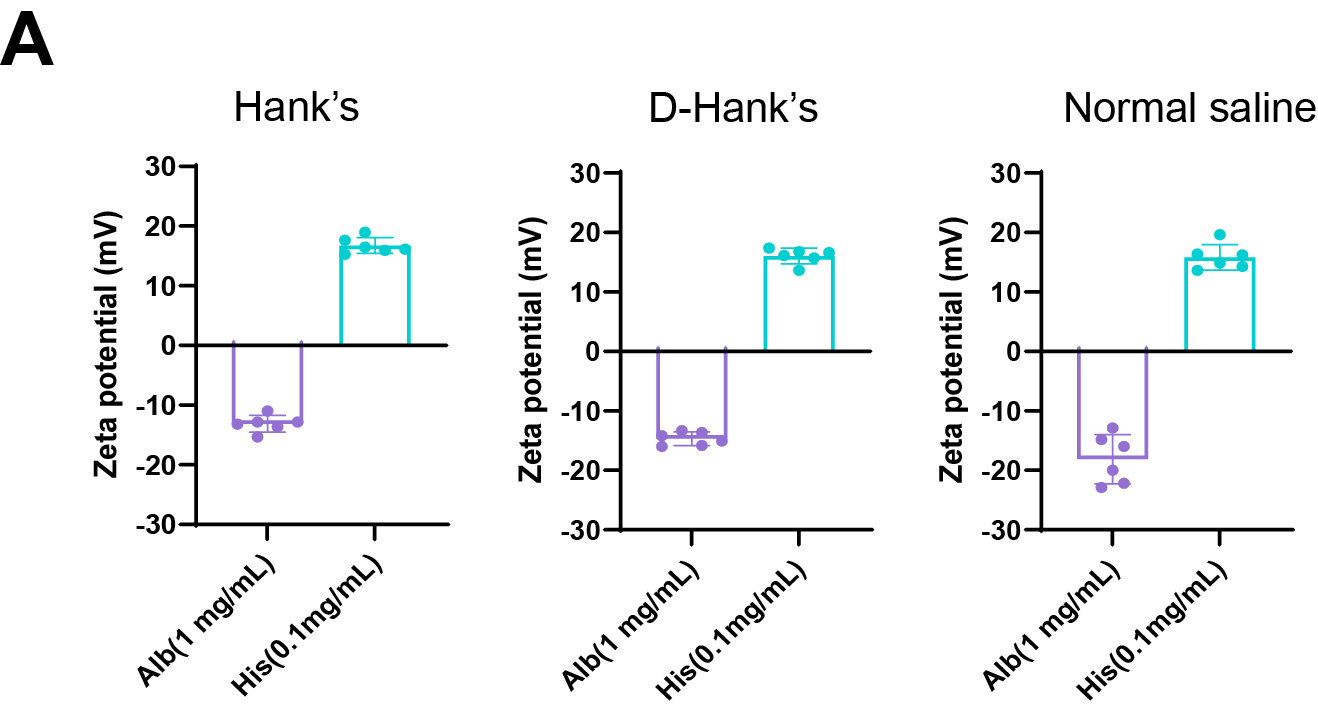


Figure S10. Zeta potential measurements of albumin and histone in various solvents.

(A) Zeta potentials of albumin and histone in Hank’s, D-Hank’s, and normal saline solutions.


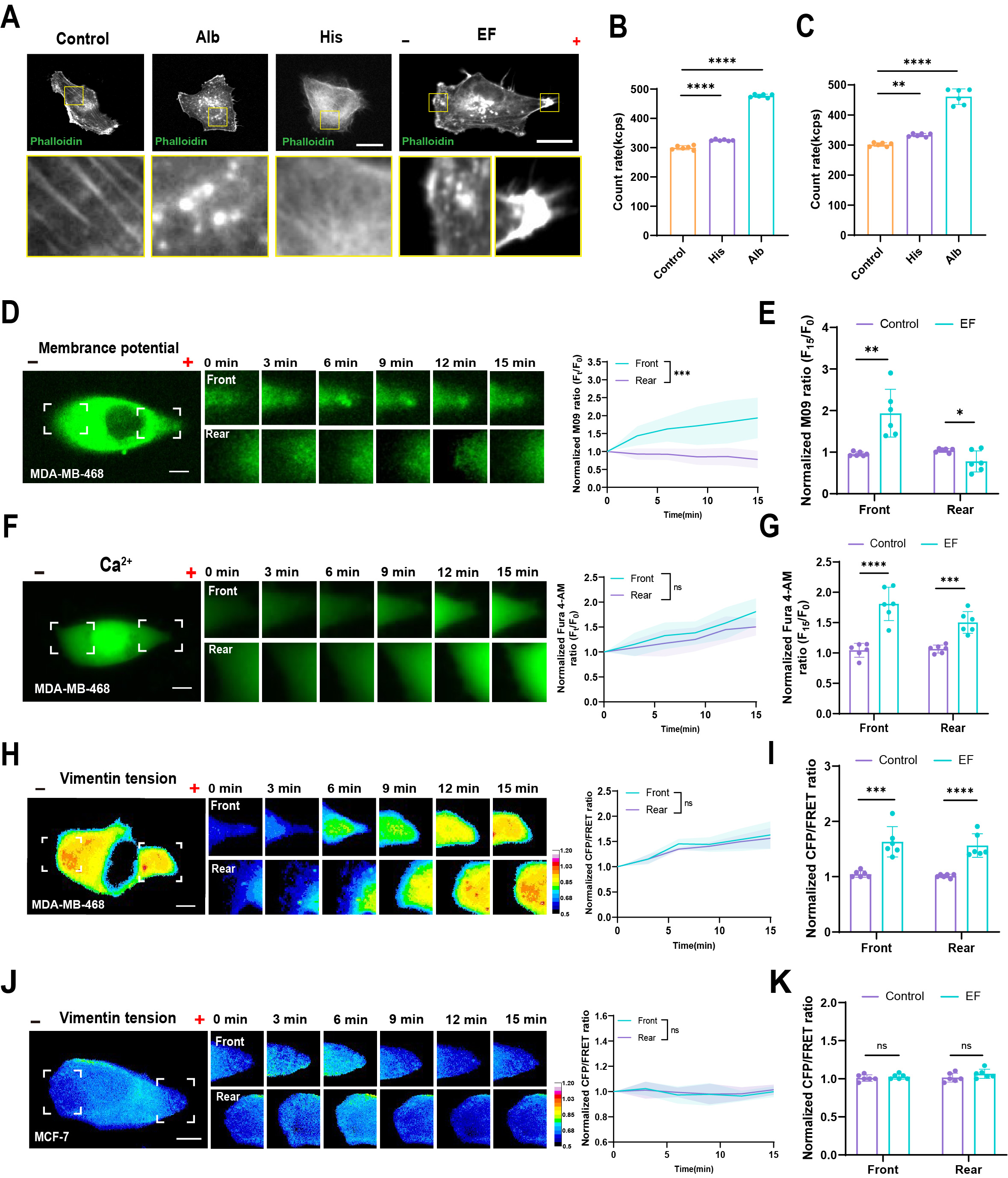
Figure S11. Electrotaxis-induced membrane potential polarization in MDA-MB-468 cells.

(A) Fluorescence images of F-actin in MDA-MB-468 cells post-treatment. Phalloidin-stained microfilament images. Scale bar: 10 μm. (B-C) Quantification of cytoplasmic protein nanoparticle (PN) numbers in MDA-MB-468 and MDA-MB-231 cells post-treatment. (mean ± SD, n ≥ 6). One-way ANOVA (***P* < 0.01, *****P* < 0.0001). (D) Left: Time-lapse images of membrane potential-sensitive dye after 15 minutes of electric field exposure (400 mV/mm). Right: Quantification of M09 fluorescence intensity at the leading and trailing edge of MDA-MB-468 cells. (mean ± SEM, n ≥ 6 cells). Unpaired Student’s t-test (****P* < 0.001). Scale bar: 10 μm. (E) Quantification of M09 fluorescence intensity at the leading and trailing edge of MDA-MB-468 cells after treatment with control and electric field. (mean ± SEM, n ≥ 6 cells). Unpaired Student’s t-test (**P* < 0.05, ***P* < 0.01). (F) Left: Time-lapse Ca²⁺ imaging under 400 mV/mm electric field stimulation (mean ± SEM, n ≥ 6). Right: Quantification of Ca²⁺ fluorescence intensity at the leading and trailing edge of MDA-MB-468 cells. (mean ± SEM, n ≥ 6 cells). Unpaired Student’s t-test (ns: not significant ). Scale bar: 10 μm. (G) Quantification of Ca²⁺ fluorescence intensity at the leading and trailing edge of MDA-MB-468 cells after treatment with control and electric field (mean ± SEM, n ≥ 6 cells). Unpaired Student’s t-test (****P* < 0.001, *****P* < 0.0001). (H, J) Left: FRET time-lapse images of Vimentin-M-cpstFRET expressing MDA-MB-468 and MCF-7 cells under 400 mV/mm stimulation. Scale bar: 10 μm. Right: Normalized CFP/FRET ratios at the leading and trailing edge of cell. (mean ± SEM, n ≥ 6 cells). Unpaired Student’s t-test (ns: not significant). (I, K) Normalized CFP/FRET ratios at the leading and trailing edge of MDA-MB-468 and MCF-7 cells after treatment with control and electric field. (mean ± SEM, n ≥ 6 cells). Unpaired Student’s t-test (****P* < 0.001, *****P* < 0.0001, ns: not significant).


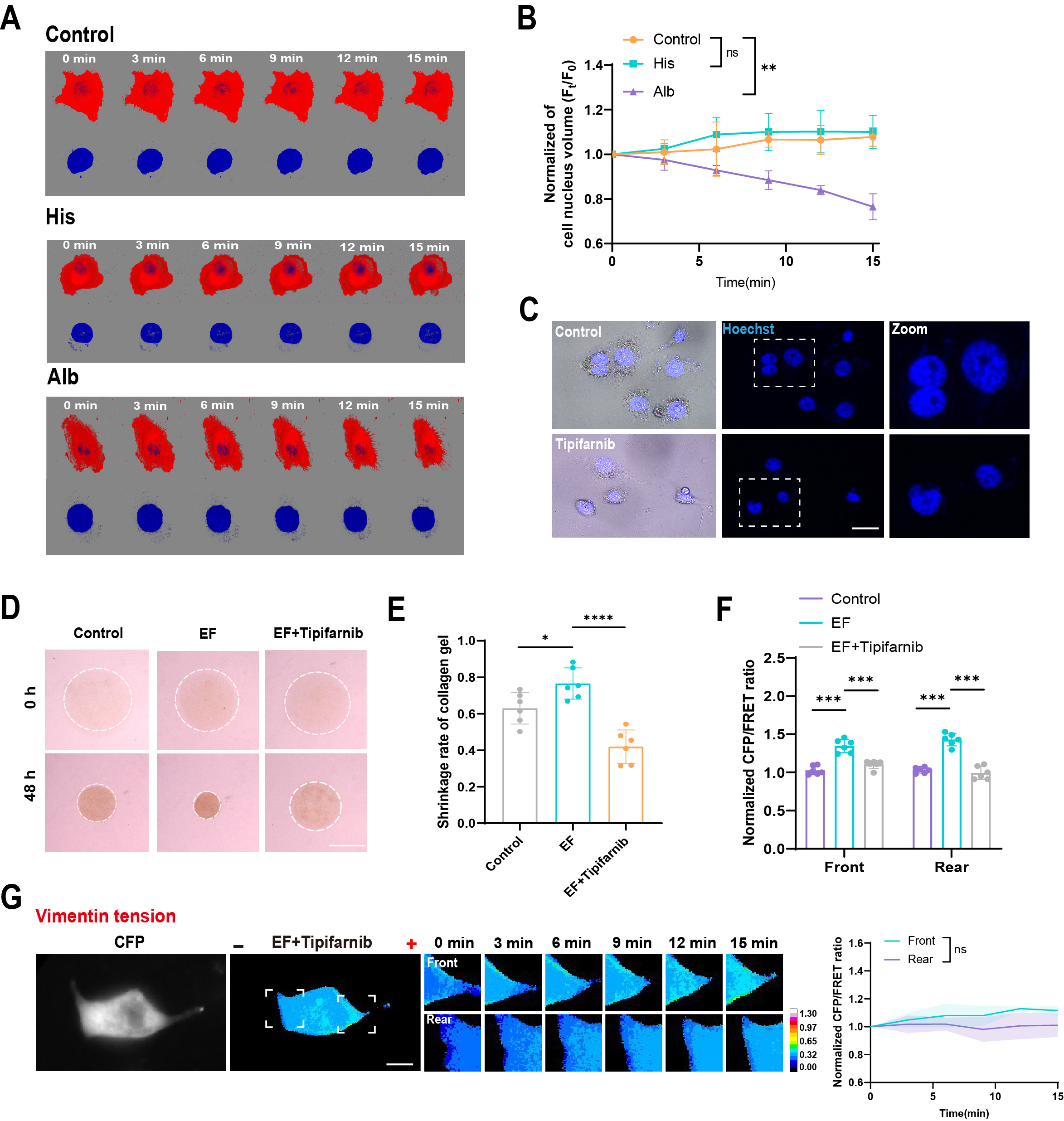


Figure S12. Regulation of electrotactic cell migration by nuclear deformation.

(A) Time-lapse images of membrane (TRITC) and nuclei (Hoechst) staining. Scale bar: 10 μm. (B) Quantified nuclear volume changes (mean ± SD, n ≥ 6). One-way ANOVA (**P < 0.01, ns: not significant). (C) Fluorescence images of nuclei (blue, Hoechst) in MDA-MB-231 cells treated with control and Tipifarnib (10 µM). Scale bar: 10 μm. (D) Contraction of collagen gels containing knockdown cells at 0 and 48 h. Scale bar: 5 mm. (E) Quantification of gel area (mean ± SD, n = 6). One-way ANOVA (**P* < 0.05, *****P* < 0.0001). (F) Normalized CFP/FRET ratios of Vimentin tension at cells front and rear after treatment with control, electric field and Tipifarnib. (mean ± SEM, n ≥ 6 cells). Unpaired Student’s t-test (****P* < 0.001). (G) Left: FRET time-lapse images of Vimentin-M-cpstFRET expressing MDA-MB-231 cells. Scale bar: 10 μm. Right: Normalized CFP/FRET ratios at the leading and trailing edge of cell. (mean ± SEM, n ≥ 6 cells). Unpaired Student’s t-test (ns: not significant).


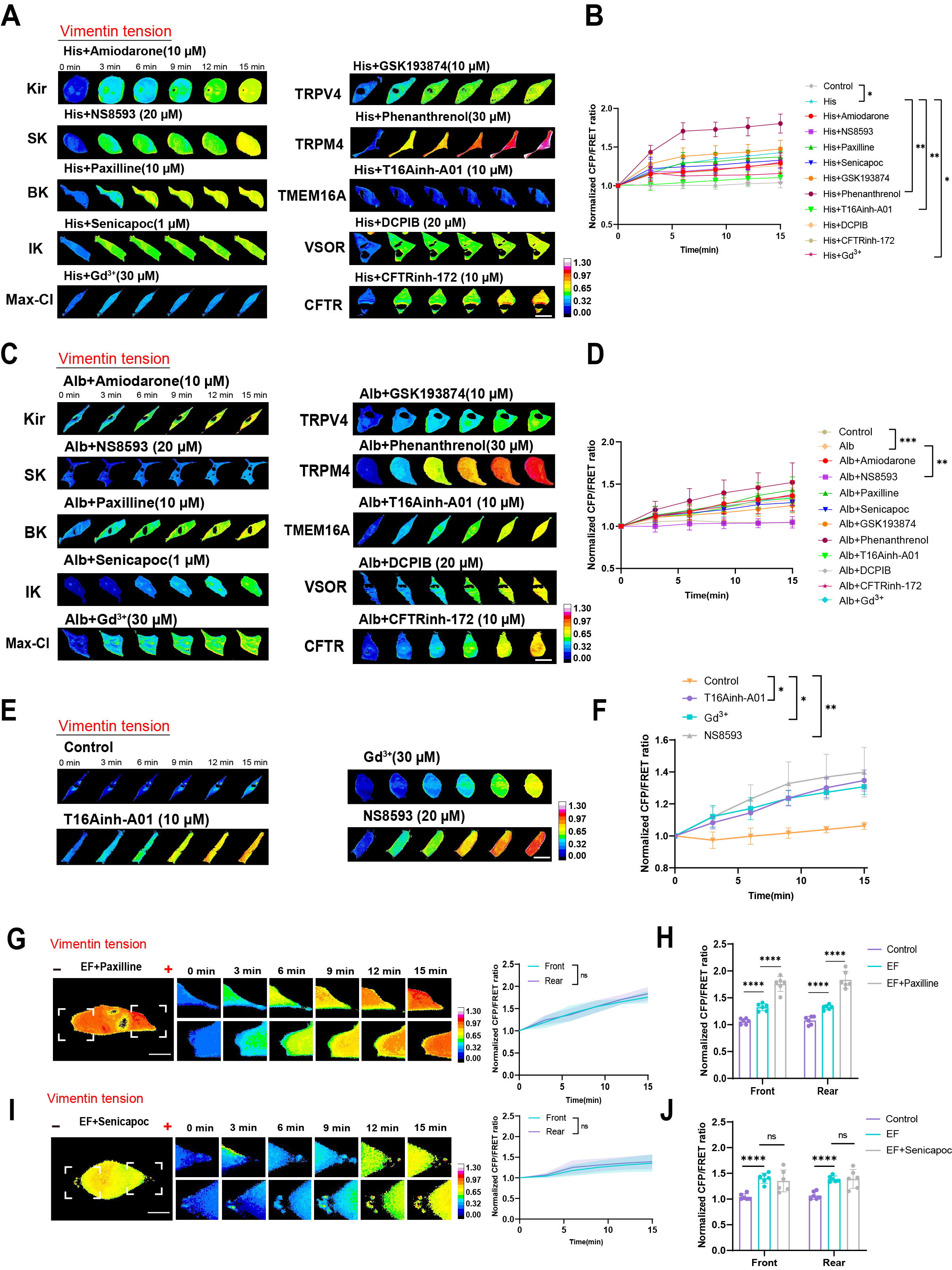


Figure S13. Screening of ion channels involved in osmotic pressure regulation in MDA-MB-231 cells.

(A-B) FRET images and CFP/FRET ratio quantification after Cl⁻ and K⁺ channel inhibition post-histone treatment. (mean ± SD, n ≥ 6). Scale bar: 10 μm. Unpaired Student’s t-test (**P* < 0.05, ***P* < 0.01). (C-D) FRET images and quantification cells after Cl⁻ and K⁺ channel inhibition post-albumin treatment. (mean ± SD, n ≥ 6). Scale bar: 10 μm. Unpaired Student’s t-test (***P* < 0.01, ****P* < 0.001). (E-F) FRET images and quantification of Vimentin-M-cpstFRET expressing cells with Max-Cl, TMEM16A, and SK channel inhibitors. (mean ± SD, n ≥ 6). Scale bar: 10 μm. One-way ANOVA (**P* < 0.05, ***P* < 0.01). (G, I) Left: FRET images of Vimentin-M-cpstFRET expressing cells after IK and BK channel inhibition under electric field stimulation. Right: Normalized CFP/FRET ratios at cells front and rear. Unpaired Student’s t-test (ns: not significant). Scale bar: 10 μm. Calibration bar: 0.00-1.30. (H, J) Normalized CFP/FRET ratios at cells front and rear after treatment with control , electric field and IK and BK channel inhibition. (mean ± SEM, n ≥ 6 cells). Unpaired Student’s t-test (ns: not significant, *****P* < 0.0001).


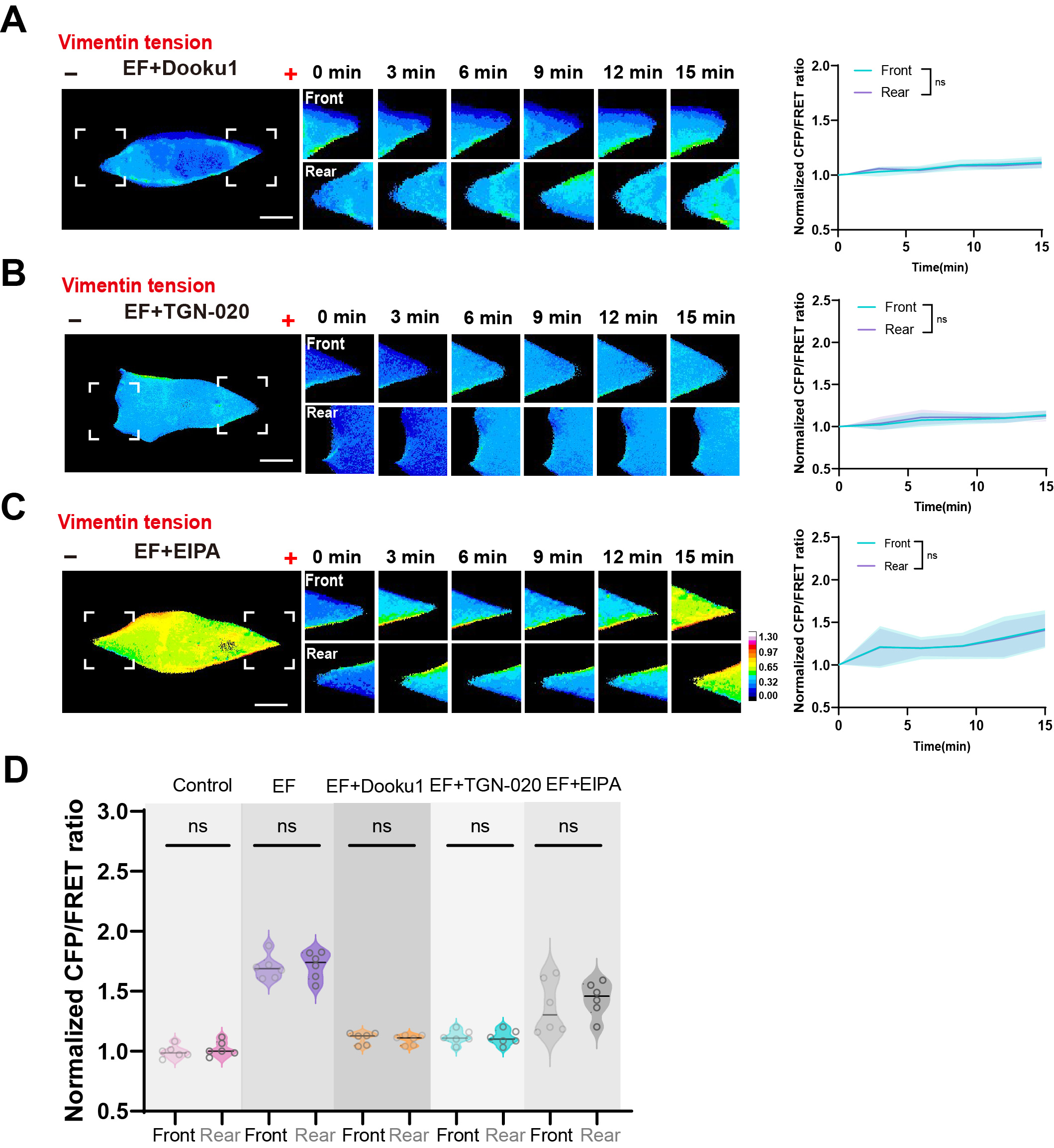


Figure S14. Regulation of cellular osmotic tension by other ion channels in electrotactic migration.

(A-C) Left, FRET images of Vimentin-M-cpstFRET expressing cells post-inhibitor treatment. Right, Normalized CFP/FRET ratios at MDA-MB-231 cells front and rear. Unpaired Student’s t-test (ns: not significant). Piezo1 inhibitor: Dooku1 (10 µM). Aquaporins inhibitor: TGN-20 (10 µM). Na^+^/H^+^ exchangers inhibitor: EIPA (10 µM). Scale bar: 10 μm. Calibration bar: 0.00-1.30. (D) Quantified front and rear tension CFP/FRET ratios (mean ± SD, n ≥ 6). Unpaired Student’s t-test (ns: not significant).


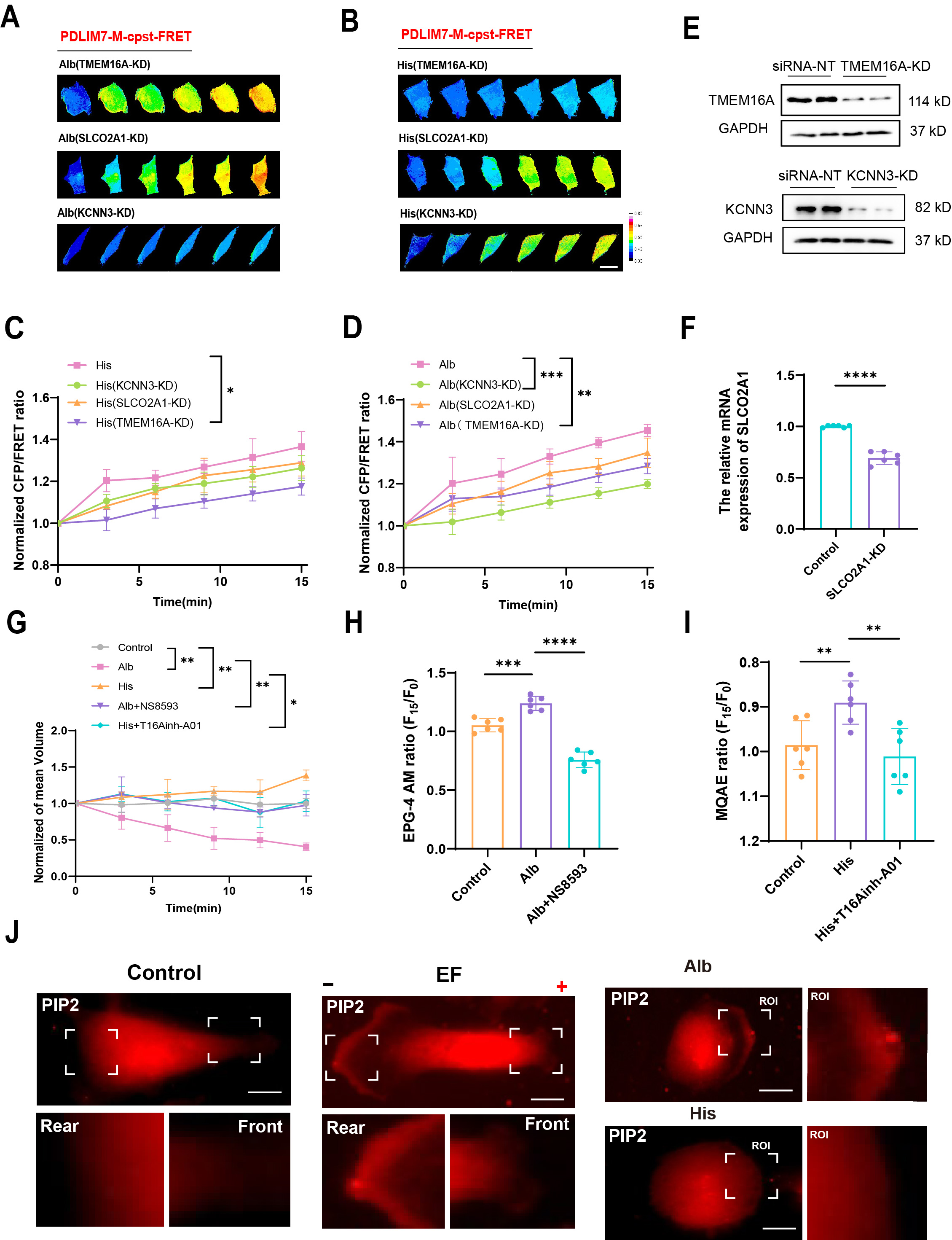


Figure S15. TMEM16A and SK regulate osmotic pressure post-histone and albumin stimulation.

(A-B) FRET images of PDLIM7-M-cpstFRET expressing cells with SLCO2A1, TMEM16A, or SK knockdown after histone or albumin treatment. (C-D) Quantification of CFP/FRET ratios post-stimulation (mean ± SD, n ≥ 6). One-way ANOVA (**P* < 0.05, ***P* < 0.01, ****P* < 0.001). (E) Immunoblot of TMEM16A and SK knockdown efficiency. (F) RT-qPCR of SLCO2A1 knockdown (mean ± SD, n = 6). Unpaired t-test (*****P* < 0.0001). (G) Cell volume measurements post-inhibitor treatment (mean ± SD, n ≥ 6). One-way ANOVA (**P* < 0.05, ***P* < 0.01). (H-I) Quantified K⁺ and Cl⁻ fluorescence intensities post-inhibitor treatment (mean ± SD, n ≥ 6). One-way ANOVA (***P* < 0.01, ****P* < 0.001, *****P* < 0.0001). (J) Immunofluorescence of PIP2 distribution (TRITC) at cell front and rear. Scale bar: 10 μm.


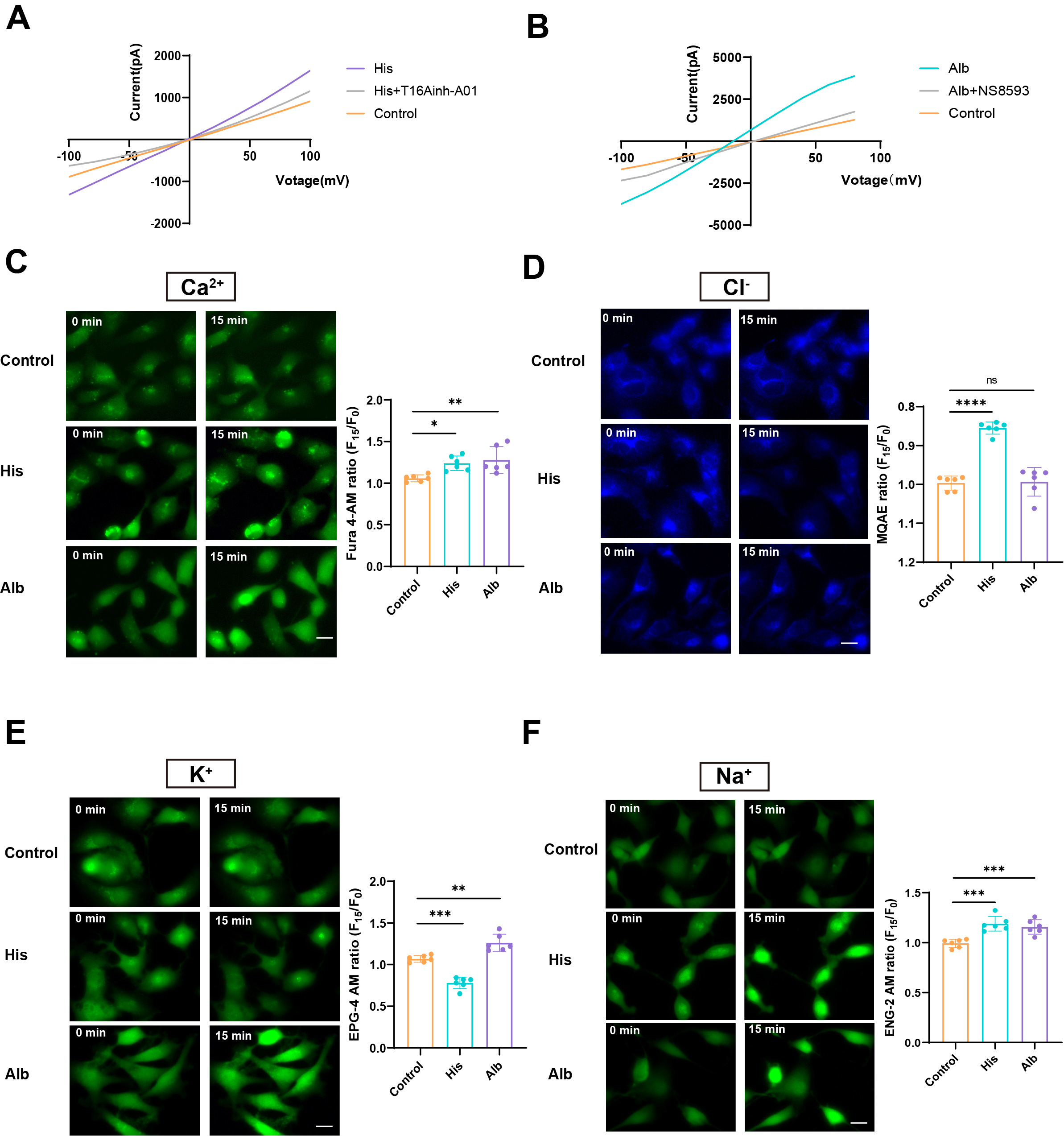


Figure S16. Electrophysiological characterization of TMEM16A and SK channels and ion dynamics under electric field stimulation.

(A-B) Current traces recorded from cells treated with TMEM16A inhibitor (T16Ainh-A01, 10 μM) and SK inhibitor (NS8593, 20 μM) under voltage step protocols. (C-F) Time-lapse imaging of intracellular Ca²⁺, Cl⁻, K⁺, and Na⁺ after treatment with histone (0.1 mg/mL) and albumin (1 mg/mL) for 15 minutes. Quantified fluorescence intensities (mean ± SD, n ≥ 6). One-way ANOVA (**P* < 0.05, ***P* < 0.01, ****P* < 0.001, *****P* < 0.0001, ns: not significant).


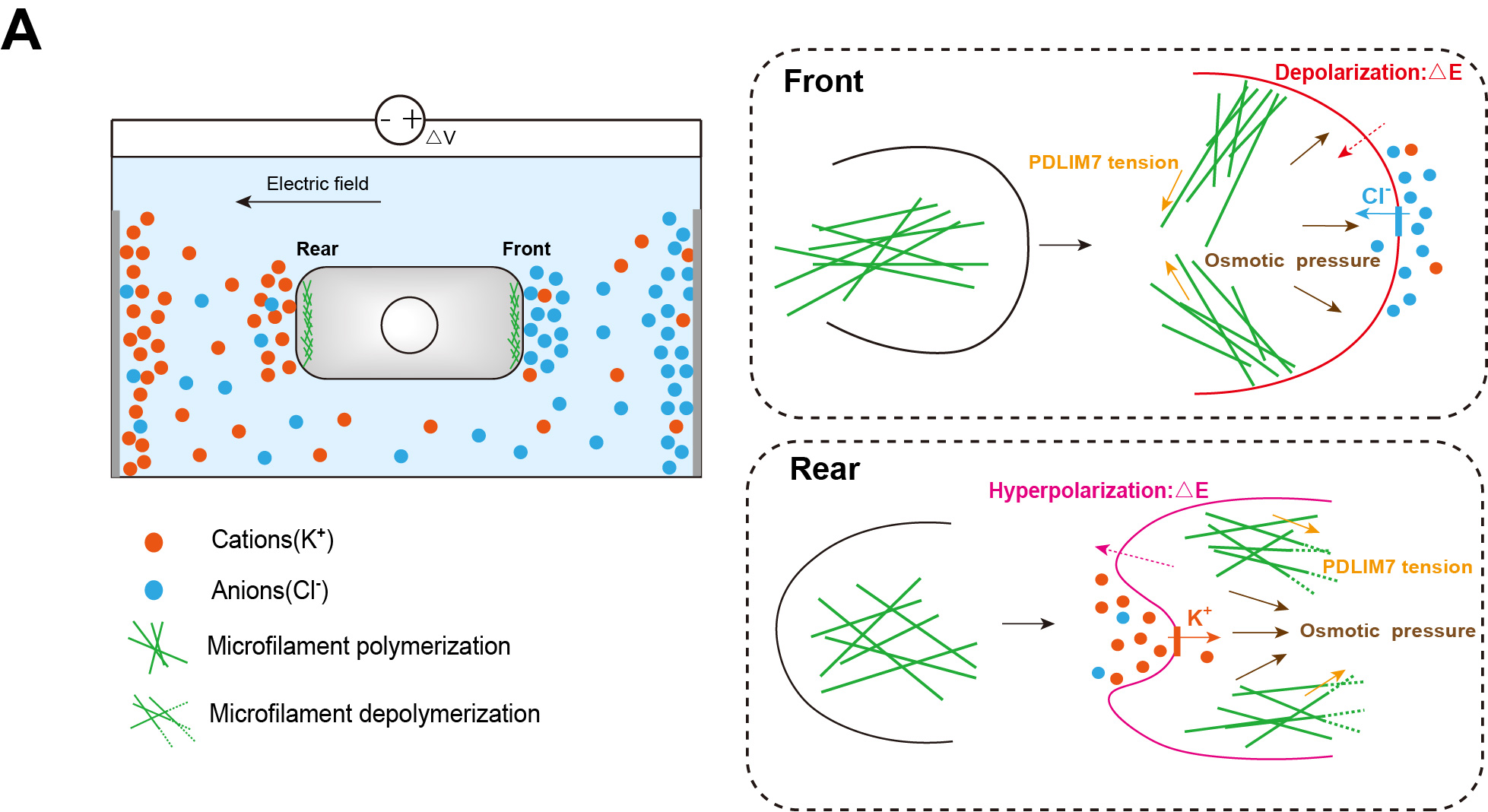


Figure S17. Schematic diagram of electrotactic cell migration.

(A) Left, cation and anion distribution at the anode- and cathode-facing sides of a cell under electric field stimulation. Right, a zoom-in schematic of the cell front and rear. Osmotic tension generates local pressure gradients within migrating cells. At the leading edge, membrane depolarization triggers chloride ion influx, which elevates intracellular osmotic pressure and drives water inflow. Inward-pulling forces through PDLIM7-microfilament tension and high OP outward-pushing forces, collectively enhancing leading-edge pressure per unit and forming expansion bulge. Conversely, at the trailing edge, membrane hyperpolarization promotes potassium efflux, which reduces osmotic pressure and promotes water efflux, leading to trailing shrinkage.
